# VIM-positive *Pseudomonas aeruginosa* in a large tertiary care hospital: matched case-control studies and a network analysis

**DOI:** 10.1186/s13756-018-0325-1

**Published:** 2018-02-27

**Authors:** Anne F. Voor in ‘t holt, Juliëtte A. Severin, Margot B. H. Hagenaars, Inge de Goeij, Diederik Gommers, Margreet C. Vos

**Affiliations:** 1 Department of Medical Microbiology and Infectious Diseases, Erasmus MC University Medical Centre, Rotterdam, The Netherlands; 2 Department of Adult Intensive Care, Erasmus MC University Medical Centre, Rotterdam, The Netherlands

**Keywords:** VIM carbapenemase, Case-control study, *Pseudomonas aeruginosa*, Network analysis, Infection prevention and control

## Abstract

**Background:**

Emergence of multidrug-resistant *Pseudomonas aeruginosa* is of global concern. We aimed to identify epidemiological relationships, the most common way of transmission, and risk factors for presence of Verona Integron-encoded Metallo-β-lactamase (VIM)-positive *P. aeruginosa* (VIM-PA).

**Methods:**

We conducted a network analysis and matched case-control studies (1:2:2). Controls were hospital-based and matched with cases for ward, day of admission (control group 1 and 2) and time between admission and the identification of VIM-PA (control group 1). The network was visualized using Cytoscape, and risk factors were determined using conditional logistic regression.

**Results:**

Between August 2003 and April 2015, 144 case patients and 576 control patients were recruited. We identified 307 relationships in 114 out of these 144 patients, with most relationships (84.7%) identified at the same department < 3 months after a previous case patient was discharged. In the multivariable model, having undergone ≥1 gastroscopy (odds ratio [OR] = 4.40, 95% confidence interval [CI] = 2.00 to 9.65 and OR = 2.47; 95% CI = 1.12 to 5.49), > 10 day use of selective digestive tract decontamination (SDD) (OR = 2.97; 95% CI = 1.02 to 8.68 and OR = 4.61; 95% CI = 1.22 to 17.37), and use of quinolones (OR = 3.29; 95% CI = 1.34 to 8.10 and OR = 3.95; 95% CI = 1.13 to 13.83 and OR = 4.47; 95% CI = 1.75 to 11.43) were identified as risk factors when using both control groups.

**Conclusions:**

The network analysis indicated that the majority of transmissions occurred on the wards, but through unidentified and presumably persistent sources, which are most likely in the innate hospital environment. Previous use of certain antibiotic regimens made patients prone to VIM-PA carriage. Additionally, gastroscopy could be considered as a high-risk procedure in patients with risk factors. Our results add to the growing body of evidence that infection control measures targeting VIM-PA should be focused on reducing antibiotics and eliminating sources in the environment.

**Electronic supplementary material:**

The online version of this article (10.1186/s13756-018-0325-1) contains supplementary material, which is available to authorized users.

## Background

The emergence of multidrug-resistant strains of *Pseudomonas aeruginosa* (MDRPA) is of global concern [1, 2]. Infections with this resistant microorganism lead to increased morbidity and mortality in patients; especially in specific patient groups, such as those in intensive care units [3–6]. MDRPA hospital outbreaks are mostly caused by MDRPA which produce carbapenemases, with as most clinically significant the metallo-β-lactamases (MBL) [2]. Currently, the Verona Integron-encoded MBL (VIM) is the most widespread MBL in *P. aeruginosa* [2, 7–9]. Sources are often hard to eradicate because *P. aeruginosa* is known to form a biofilm in environmental niches which protects it from cleaning and disinfection actions [10, 11].

Since 2003, a VIM-positive clone of *P. aeruginosa* (VIM-PA) has emerged in our hospital and became entrenched causing multiple episodes of colonizations and infections in patients [9, 12]. A systematic review published by our research group showed that the leading risk factors for acquiring MDRPA were carbapenem use and having medical devices [13]. However, risk factors are likely to be outbreak specific because of different local circumstances and patient populations.

The aim of this study was first to identify epidemiological relationships between patients with a VIM-PA, and to identify the most common way of transmission. Second, we aimed to identify risk factors for presence of VIM-PA among colonized and/or infected patients with a case-control study. When a case-control study is used to understand an outbreak, it is often not clear what the best control group is. Both under- and overmatching may affect the results; in essence, the choice of the control determines the outcome. Therefore, our third aim was identifying the most appropriate control group.

## Methods

### Ethics statement

Written approval to conduct this study was received from the medical ethics research committee of the Erasmus MC University Medical Centre (Erasmus MC), Rotterdam, the Netherlands (MEC-2015-240). This study is registered in the Dutch National Trial Register (NTR5145).

### Setting

This retrospective study was conducted at the Erasmus MC in Rotterdam, the Netherlands, using data from August 2003 until April 2015. In this 1200-bed university hospital all medical specialties are available; organized into 48 departments. The Department of Adult Intensive Care (adult ICU) comprises of three high-level ICU wards, and each ward has only single-patient rooms. At the ICU, patients expected to be on a mechanical ventilator for > 48 h or anticipated to be admitted to the ICU for > 72 h receive selective digestive tract decontamination (SDD). The SDD regimen is identical to the regimen used by de Smet et al., including 4 days of cefotaxime intravenously [14]. The total number of clinical admissions and clinical admission days from 2003 until 2015 are available in Additional file 1.

### Patient inclusion and microbiological analysis

Patients were included if identified with VIM-PA between 48 h after admittance to and 48 h after discharge from a department in the main Erasmus MC building. Patients were excluded if admitted only to the Erasmus MC Sophia Children’s Hospital or only to the Erasmus MC Cancer Institute. These buildings are physically separated from the main building, and have their own employees. To our knowledge, there has been no cross-over of VIM-PA to and from these separate buildings. In addition, 22 patients that were involved in an outbreak resulting from a contaminated duodenoscope used for endoscopic retrograde cholangiopancreatography were excluded. The exact cause, source and transmission route were known and it was therefore investigated and reported separately [15].

Cultures taken for clinical diagnostic purposes were processed in the laboratory using standard microbiological methods. In case of suspected growth of carbapenemase-producing *P. aeruginosa* or MDRPA, an in-house polymerase chain reaction (PCR) for detection of *bla*_VIM_ on LightCycler 480 (Roche Diagnostics, Almere, The Netherlands) was performed using previously reported primers [9, 12]. For screening for VIM-PA, swabs were obtained from throat and rectum, cultured overnight at 35 °C in a Tryptic Soy Broth with ceftazidime (2 mg/L) and vancomycin (50 mg/L), followed by our in-house PCR test on the broth. Positive PCR results were confirmed by subculturing the broth on a blood agar (BD Diagnostics, Breda, The Netherlands); *P. aeruginosa* growing on this agar plate was subjected to *bla*_VIM_ PCR. Identification and susceptibility testing was performed using Vitek2 (bioMérieux, Marcy l’Etoile, France). Since January 2013, the MALDI-TOF (Bruker Daltonics, Bremen, Germany) was used for identification. Clonal relatedness of VIM-PA from clinical and screening cultures was determined using the DiversiLab system with the *Pseudomonas* kit (bioMérieux).

General infection prevention and control measures were installed after each case was identified (e.g. isolation). However, in 2011 these measures were intensified; at two adult intensive care units (ICUs), twice-weekly screening for VIM-PA (i.e. rectum and throat cultures) was implemented from October 2011. However, after April 2014, this was reduced to once a week. After August 2014, the weekly screening halted; however, was re-implemented in September 2014 because a new case of VIM-PA was identified from a clinical sample. Additionally, at the ICU rectum and throat cultures on VIM are taken upon admittance and discharge of patients. At the neurological high care ward, screening took place once a week from August 2013 until January 2016.

### Network analysis

Admission histories in time and department/room location of patients identified with VIM-PA were retrieved to define epidemiological relatedness. Each identified relation was classified in one out of four categories (Table 1). Then, the data were imported into Cytoscape v3.2.1 (http://www.cytoscape.org) and the network was visualized [16]. It was analysed whether a patient only ‘received’, only ‘transmitted’, or ‘received and transmitted’ the VIM-PA following the definitions in Table 1. “Only received” indicated that a patient did not have epidemiological links to patients identified with a VIM-PA at a later time, “only transmitted” indicated that a patient did not have epidemiological links to patients identified with a VIM-PA earlier in time. “Received and transmitted” indicated that a patient had epidemiological relationships to patients identified with VIM-PA earlier in time and later in time.

**Table 1:** Definitions of epidemiological relatedness

	Definite^1^	Probable	Possible-I	Possible-II	Impossible
Same patient room	1	1	0	0	0
Same department	1	1	1	1	0
Same period	1	0^a^	1	0^a^	0^a^/0/1

0 = no; 1 = yes, 0^a^ = < 3 months after previous positive patient was discharged. ^1^Definition definite was not possible at the intensive care units because only single patient rooms are present

### Case-control studies

The risk factor analysis was performed in individual matched retrospective case-control studies, using a 1:2:2 ratio, with hospital-based controls. All information was extracted from the electronic medical records. A list of all patient and treatment related variables collected for cases and controls is presented in Additional file 2.

#### Control groups

Patients in control group 1 and 2 were matched for the following three characteristics: I: admitted to the same ward where the case supposedly acquired the VIM-PA (i.e. the ward where the patient was admitted 48 h before the positive culture) (exact match), II: being admitted on the same date as the case (best match), III: having the same days of exposure as the case (i.e. the days between admittance and the date of the first positive culture with VIM-PA) (best match). If exact matching was not possible ─with the exception of ward─ exposure time was found to be the most imperative factor. Patients in control group 3 and 4 were matched for the following characteristics: I: admitted to the same ward as the case (exact match), II: admittance on the same date as the case (best match). The control patient had to be free of colonization or infection with VIM-PA. This could be proven either by negative screening cultures or by the absence of clinical cultures with VIM-PA. A control patient could not serve as a control more than two times; within and between the four different control groups. Also, a case patient could never be selected as a control patient.

#### Statistical analyses

For continuous variables, means or medians were calculated. For categorical variables, percentages were calculated. The conditional logistic regression model was used in both univariate and multivariable analyses. Univariate analyses were conducted using the COXREG procedure in SPSS version 21 (IBM Corp., Armonk, New York, USA). Characteristics with a *P*-value of < 0.1 in univariate analysis were included in the multivariable analyses. Treatment variables could be included as 1) use yes/no, 2) use for 0/1–3/≥4 days or 3) use for 0/1-3/4-10/≥11 days. Selection for inclusion in the multivariable model of either category 1, 2 or 3 of a certain antibiotic was based on: 1) > 5 patients in present in each group, 2) estimates of the different categories had to show a difference of at least 1 odds ratio (OR). Multivariable analyses were conducted using conditional logistic regression with dynamic ridge penalties in the R Project for statistical computing version 3.3.1 (Vienna, Austria). Subgroup analyses were performed for ward of acquisition of case patients and matched controls being ICU or being non-ICU, as well as for case patients in clonal clusters as indicated by the typing results. Additionally, analyses were performed between patients in control group 1&2 and 3&4. Results were presented as ORs with 95% confidence intervals (CI). *P*-values < 0.05 were considered statistically significant. Graphs were created using GraphPad Prism Version 7.01 (GraphPad Software, Inc. CA, USA).

## Results

### Included patients

Out of 166 patients identified with a VIM-PA between August 2003 and April 2015, eight children were excluded because they were admitted only to the Erasmus MC Sophia Children’s hospital and one patient was excluded because admitted only to the Erasmus MC Cancer Institute. In addition, 13 patients were excluded because the VIM-PA was identified within 48 h after admission. Ultimately, 144 patients were included in the network analysis and as case patients in the case-control study (Additional file 1). Nineteen different wards of acquisition were identified, including three ICUs (i.e. two general adult ICUs and 1 thoracic ICU). The top five locations of acquisition were the two general adult ICUs (87 patients, 60.4%), the gastro-intestinal surgical ward (10 patients; 6.9%), and the gastroenterology and hepatology ward (7 patients, 4.9%). Typing showed that the VIM-PA of 29 (20.1%) patients belonged to clonal cluster A, 105 (72.9%) to clonal cluster B, 7 (4.9%) did not belong to clonal cluster A or B, and it was not possible to type strains from three patients (2.1%).

### Network analysis

**Fig. 1:**
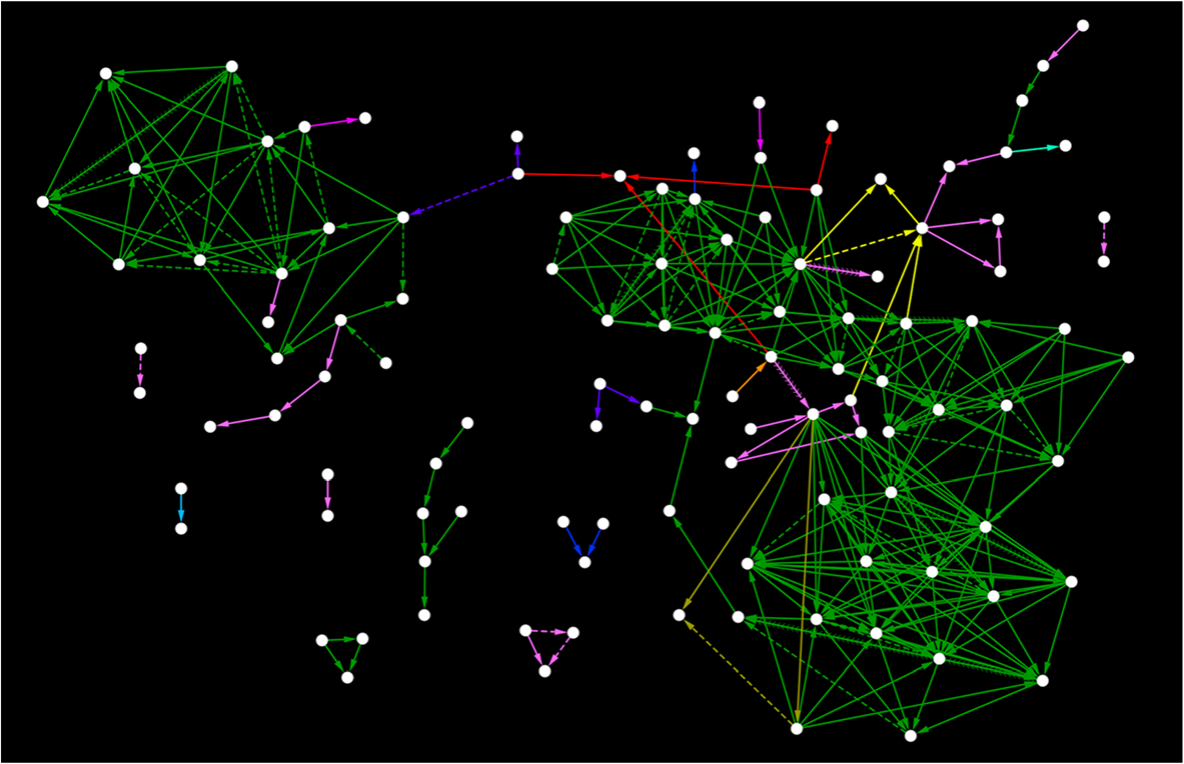
Network of 307 relationships in 114 out of 144 patients identified with VIM-PA. Thirty out of 144 patients did not have relationships to other case patients (20.8%). Edge colours represent different Erasmus MC departments; green represents the two adult ICU wards. Line shapes represent the different epidemiological relationships as described in Table 1: contiguous line = probable, dash line = possible A, solid line = possible B. The arrow shows the direction of the relationship

From 2003 until 2015, we identified 307 relationships in 114 out of 144 patients (Table 1, Fig. 1). Thirty out of 144 patients did not have relationships to other case patients (20.8%). When considering the definitions (Table 1) we identified nine probable relationships, 38 possible-I relationships and 260 possible-II relationships at 12 different departments. Most relationships (92%) were identified at the ICU. Twenty-five patients (17.4%) only ‘received’ the VIM-PA, 22 (15.3%) only ‘transmitted’ VIM-PA and 67 (46.5%) ‘received and transmitted’ VIM-PA.

### Case-control studies

#### Matching

It was impossible to perfectly match all cases to four controls. Overall, perfect matching was achieved in 38.2% (range: 16.0%–66.0%). For cases, the median days from admission to acquisition of VIM-PA was 14 days (range: 1–114 days). In the control groups, the median error in days was 4, 4, − 1 and − 1 days respectively. Seventeen patients served two times as control patient between the four control groups (11.8%).

#### Risk factors for acquisition

**Fig. 2:**
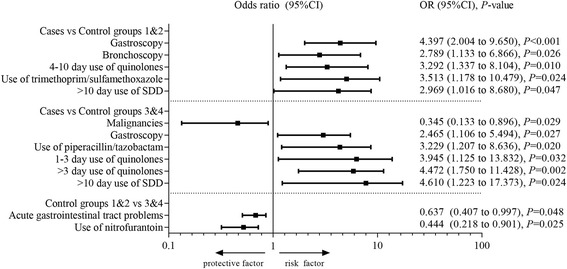
Risk factors and protective factors identified using multivariable analysis. Abbreviations: SDD = selective digestive tract decontamination, 95%CI = 95% confidence interval

Patient related clinical variables with crude odds ratios, 95% CI and *P*-values are presented in Table 2. Compared to control group 1&2 and control group 3&4, the median length of admission was significantly longer and the 1-year mortality rate was significantly higher in case patients (Table 2). Treatment related variables with crude odds ratios, 95% CI and *P*-values are presented in Table 3 and in Additional file 3. When comparing cases to control group 1&2, multivariable analysis revealed five risk factors; two patient related clinical risk factors, and three treatment related risk factors (Fig. 2). The highest odds ratio was identified for having undergone ≥1 gastroscopy 6 months prior to the identification of VIM-PA (OR = 4.40, 95%CI = 2.00 to 9.65, *P* < 0.001). When comparing cases to control group 3&4, one patient related clinical protective factor was identified (i.e. malignancies) and one risk factor (i.e. gastroscopy) (Fig. 2). Also, four treatment related risk factors were identified; use of piperacillin/tazobactam, 1–3 day and > 3 day use of quinolones and > 10 day use of SDD (Fig. 2). The highest odds ratio was identified for > 10 day use of SDD (OR = 4.61, 95%CI = 1.22 to 17.37, *P* = 0.024). In both case-control studies, previous use of quinolones, use of SDD for > 10 days, and having undergone ≥1 gastroscopy 6 months prior to the identification of VIM-PA were identified as risk factors, and could therefore be considered robust risk factors.

**Table 2:** Patient related clinical variables, univariate analyses between cases and controls 1&2 and between cases and controls 3&4

Variables	Cases (*n* = 144)	Controls 1&2 (*n* = 288)	Crude OR (95% CI)	*P*-value	Controls 3&4 (*n* = 288)	Crude OR (95% CI)	*P*-value
Basic characteristics							
Age, years, median (range)	58.3 (17–82)	60.3 (17–92)	0.998 (0.986–0.1010)	0.723	61.1 (17–91)	0.995 (0.982–1.007)	0.407
Male gender (%)	83 (57.6)	174 (60.4)	0.886 (0.584–1.345)	0.570	167 (58.0)	0.985 (0.650–1.493)	0.944
28-day mortality (%)	41 (28.5)	69 (24.0)	1.283 (0.804–2.048)	0.296	40 (13.9)	**2.677 (1.577–4.544)**	**< 0.001**
1-year mortality (%)	82 (56.9)	95 (33.0)	**2.680 (1.754–4.094)**	**< 0.001**	63 (21.9)	**4.950 (3.072–7.976)**	**< 0.001**
Transferred from another hospital (%)	50 (34.7)	60 (20.8)	**2.000 (1.279–3.128)**	**0.002**	50 (17.4)	**2.405 (1.530–3.782)**	**< 0.001**
Median length of admission (range)	55 (3–338)	25 (1–258)	**1.021 (1.014–1.028)**	**< 0.001**	12 (1–1102)	**1.021 (1.014–1.027)**	**< 0.001**
Erasmus MC 1y before VIM-PA (%)	64 (44.4)	111 (38.5)	1.278 (0.851–1.920)	0.237	109 (37.8)	1.348 (0.881–2.064)	0.169
Erasmus MC ICU 1y before VIM-PA (%)	14 (9.7)	15 (5.2)	1.867 (0.901–3.867)	0.093	14 (4.9)	2.131 (0.976–4.652)	0.058
Surgery (%)	87 (60.4)	119 (41.3)	**2.165 (1.432–3.275)**	**< 0.001**	121 (42.0)	**2.119 (1.399–3.209)**	**< 0.001**
Underlying diseases							
Cystic fibrosis (%)	2^a^ (1.4)	3 (1.0)	NA	NA	2 (0.7)	NA	NA
Chronic respiratory illness (%)	29 (20.1)	43 (14.9)	1.488 (0.861–2.571)	0.155	43 (14.9)	1.518 (0.868–2.655)	0.144
Chronic kidney failure (%)	5 (3.5)	3 (1.0)	3.33 (0.797–13.948)	0.099	11 (3.8)	0.903 (0.304–2.689)	0.855
Acute kidney failure; use of CVVH (%)	28 (19.4)	18 (6.3)	**3.471 (1.844–6.533)**	**< 0.001**	10 (3.5)	**6.651 (3.022–14.638)**	**< 0.001**
Chronic liver failure (%)	5 (3.5)	7 (2.4)	1.480 (0.442–4.952)	0.525	17 (5.9)	0.588 (0.217–1.594)	0.297
Acute liver failure (%)	0 (0)	6 (2.1)	NA	NA	3 (1.0)	NA	NA
Chronic problems of the gastrointestinal tract (%)	19 (13.2)	28 (9.7)	1.377 (0.758–2.502)	0.294	26 (9.0)	1.603 (0.822–3.126)	0.166
Acute problems of the gastrointestinal tract (%)	28 (19.4)	39 (13.5)	1.493 (0.894–2.494)	0.126	54 (18.8)	1.047 (0.627–1.750)	0.861
Auto-immune disease (%)	7 (4.9)	18 (6.3)	0.762 (0.308–1.886)	0.556	26 (9.0)	0.520 (0.221–1.223)	0.134
Human immunodeficiency virus (%)	0 (0)	2 (0.7)	NA	NA	4 (1.4)	NA	NA
Diabetes (%)	24 (16.7)	37 (12.8)	1.353 (0.776–2.360)	0.287	48 (16.7)	1.000 (0.579–1.726)	1.000
Solid organ transplant recipient (%)	20^a^ (14.0)	20 (6.9)	**2.257 (1.139–4.473)**	**0.020**	30 (10.4)	1.463 (0.784–2.729)	0.232
Stem cell/bone marrow transplant recipient (%)	7^a^ (4.9)	6 (2.1)	3.562 (0.875–14.493)	0.076	1 (0.3)	NA	NA
Use of immunosuppressive agents (%)	55 (38.2)	71 (24.7)	**1.920 (1.236–2.985)**	**0.004**	55 (19.1)	**2.493 (1.602–3.879)**	**< 0.001**
Malignancies (%)	32 (22.2)	76 (26.4)	0.777 (0.472–1.280)	0.322	92 (31.9)	**0.570 (0.347–0.936)**	**0.026**
Neutropenia, < 500 cells/μL (%)	3^b^ (2.7)	4^b^ (2.1)	0.883 (0.159–4.889)^b^	0.886	7^b^ (2.4)	0.667 (0.164–2.713)^b^	0.571
Endoscopies							
Colonoscopy (%)	7 (4.9)	10 (3.5)	1.400 (0.533–3.678)	0.495	12 (4.2)	1.202 (0.432–3.345)	0.724
Sigmoidoscopy (%)	7 (4.9)	5 (1.7)	2.800 (0.889–8.822)	0.079	8 (2.8)	1.750 (0.635–4.826)	0.280
Endoscopic ultrasound (%)	7 (4.9)	26 (9.0)	0.490 (0.202–1.189)	0.115	19 (6.6)	0.684 (0.260–1.802)	0.443
Gastroscopy (%)	75 (52.1)	50 (17.4)	**6.609 (3.803–11.485)**	**< 0.001**	59 (20.5)	**4.529 (2.79–7.335)**	**< 0.001**
ERCP (%)	17 (11.8)	15 (5.2)	**2.520 (1.190–5.336)**	**0.016**	10 (3.5)	**3.917 (1.678–9.143)**	**0.002**
Bronchoscopy (%)	41 (28.5)	34 (11.8)	**3.159 (1.841–5.422)**	**< 0.001**	33 (11.5)	**3.030 (1.800–5.103)**	**< 0.001**
Transoesophageal Echocardiography (TEE) (%)	12 (8.3)	13 (4.5)	1.846 (0.842–4.046)	0.126	13 (4.5)	1.901 (0.849–4.257)	0.118
Medical devices							
Mechanical ventilation (%)	129 (89.6)	210 (72.9)	**4.921 (2.284–10.600)**	**< 0.001**	185 (64.2)	**8.211 (3.857–17.481)**	**< 0.001**
Tracheostomy (%)	29 (20.1)	38 (13.2)	**1.801 (1.011–3.211)**	**0.046**	9 (3.1)	**7.883 (3.443–18.045)**	**< 0.001**
Extracorporeal membrane oxygenation (%)	2 (1.4)	0 (0)	NA	NA	0 (0)	NA	NA
Central venous catheter (%)	103 (71.5)	124 (43.1)	**4.121 (2.477–6.854)**	**< 0.001**	77 (26.7)	**9.040 (5.101–16.023)**	**< 0.001**

*Abbreviations*: *VIM-PA* Verona Integron-encoded Metallo-β-lactamase (VIM)-positive *Pseudomonas aeruginosa*, *95% CI* 95% confidence interval, *y* year, *ICU* intensive care unit, *CVVH* Continuous Veno-Venous Hemofiltration, *ERCP* Endoscopic Retrograde Cholangiopancreatography

^a^1 case missing because no medical background was available

^b^For 32 cases, 99 controls 1&2 and 108 controls 3&4 no information about neutrophils was available

bold = statistically significant

**Table 3:** Treatment related variables, univariate analyses between cases and controls 1&2 and between cases and controls 3&4

Variables^c^	Cases (*n* = 138^a^)	Controls 1&2 (*n* = 288)	Crude OR (95% CI)	*P*-value	Controls 3&4 (*n* = 288)	Crude OR (95% CI)	*P*-value
Antifungals (%)	82 (59.4)	79 (28.0)^b^	**4.863 (2.886–8.194)**	**< 0.001**	50 (17.7)^b^	**5.890 (3.647–9.512)**	**< 0.001**
Antivirals (%)	16 (11.6)	19 (6.7)^b^	1.913 (0.899–4.071)	0.092	17 (6.0)^b^	**2.053 (1.011–4.169)**	**0.047**
Aminoglycosides (%)	51 (37.0)	53 (18.8)	**2.988 (1.769–5.046)**	**< 0.001**	24 (8.5)	**6.832 (3.692–12.643)**	**< 0.001**
Amoxicillin/clavulanic acid (%)	39 (29.1)	48 (17.0)^b^	**2.014 (1.196–3.394)**	**0.009**	40 (14.2)^b^	**2.388 (1.428–3.992)**	**0.001**
Carbapenems (%)	58 (42.0)	58 (20.7)	**3.124 (1.898–5.142)**	**< 0.001**	26 (9.2)	**6.762 (3.810–12.000)**	**< 0.001**
Cephalosporins (%)	104 (77.6)	131 (46.5)	**3.942 (2.383–6.520)**	**< 0.001**	107 (37.9)	**6.407 (3.692–11-121)**	**< 0.001**
Colistin (%)	17 (12.7)	20 (7.1)^b^	1.896 (0.926–3.880)	0.080	12 (4.3)^b^	**4.282 (1.756–10.437)**	**0.001**
Macrolides (%)	60 (44.8)	64 (22.7)	**2.702 (1.696–4.304)**	**< 0.001**	27 (9.6)	**6.881 (3.885–12.190)**	**< 0.001**
Metronidazole (%)	48 (35.8)	48 (17.0)	**2.445 (1.534–3.898)**	**< 0.001**	32 (11.3)^b^	**4.689 (2.630–8.360)**	**< 0.001**
Nitrofurantoin (%)	17 (12.7)	12 (4.3)^b^	**3.486 (1.543–7.878)**	**0.003**	24 (8.5)	1.527 (0.767–3.039)	0.228
Penicillin (%)	29 (21.6)	49 (17.4)	1.226 (0.739–2.032)	0.431	31 (11.0)	**2.100 (1.199–3.678)**	**0.009**
Piperacillin/tazobactam (%)	49 (36.6)	50 (17.7)^b^	**2.774 (1.666–4.621)**	**< 0.001**	32 (11.3)^b^	**4.572 (2.604–8.025)**	**< 0.001**
Quinolones (%)	94 (70.1)	88 (31.2)	**6.087 (3.553–10.429)**	**< 0.001**	53 (18.8)	**9.193 (5.284–15.996)**	**< 0.001**
Trimethoprim/sulfamethoxazole (%)	32 (23.9)	23 (8.2)^b^	**3.606 (1.930–6.740)**	**< 0.001**	19 (6.7)^b^	**3.834 (2.098–7.007)**	**< 0.001**
Vancomycin (%)	74 (55.2)	51 (18.1)	**6.355 (3.652–11.057)**	**< 0.001**	27 (9.6)	**9.847 (5.437–17.836)**	**< 0.001**
Other antibiotics (%)	32 (23.9)	28 (9.9)^b^	**3.074 (1.680–5.627)**	**< 0.001**	30 (10.6)^b^	**2.684 (1.512–4.763)**	**0.001**
Selective digestive tract decontamination (%)	92 (68.7)	109 (38.7)	**4.583 (2.641–7.952)**	**< 0.001**	59 (20.9)	**10.864 (5.748–20.536)**	**< 0.001**

*Abbreviations*: *95% CI* 95% confidence interval, ^a^Data on antibiotics of the first six cases was missing, bold = statistically significant, *P*-value < 0.05. ^b^Included in multivariable analysis. ^c^For categorical data see Additional file 3

**Fig. 3:**
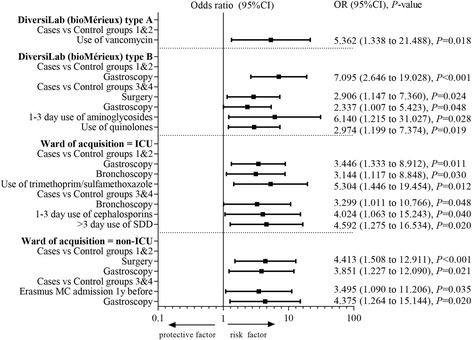
Risk factors identified using multivariable analysis of subgroups DiversiLab type A or type B and ward of acquisition of the case patient being the intensive care unit (ICU) or non-ICU wards. Abbreviations: SDD = selective digestive tract decontamination, 95%CI = 95% confidence interval, ICU = intensive care unit

Univariate results of the subgroup analyses are presented in Additional files 4 and 5. Multivariable results are displayed in Fig. 3. There are differences in identified risk factors in the different subgroups. For example, for patients with DiversiLab type A endoscopies did not seem to play a role, whereas in type B they did. Also, at the ICU antibiotic use (trimethoprim/sulfamethoxazole) was identified as a risk factor in combination with having undergone ≥1 gastroscopy and bronchoscopy 6 months prior to the identification of VIM-PA, whereas at non-ICU wards it was a combination of having undergone ≥1 gastroscopy 6 months prior to the identification of VIM-PA and surgery or being admitted at the Erasmus MC before.

Univariate differences between control group 1&2 and 3&4 are presented in Additional file 6. Multivariable analysis revealed only two differences between the control groups, regarding protection by acute gastrointestinal tract problems and use of nitrofurantoin (Fig. 2).

## Discussion

Our study aimed to identify epidemiological relationships, the most common way of transmission and risk factors for presence of VIM-PA. In the network analysis, we did not identify definite relationship and only nine probable relationships. Therefore, the same patient room, either sharing a patient room or being admitted at the same patient room within 3 months, is not the most likely source. However, there was a relation with the same department. Surprisingly, the same admission period seems not to be important; most relationships were identified within 3 months after the previous positive patient was discharged. Thus, the majority of transmissions occurred on the wards in a wide time frame. Therefore, it must have occurred through unidentified sources, which may be either undetected patients or unidentified sources in the innate environment. Given the fact that patients at the ICUs and neurology high-care ward were frequently screened; we assume that undetected patients are not plausible. Our hypothesis is that persistent sources in the innate environment play an important role in the route of transmission of this pathogen. This is in agreement with current knowledge on the behaviour of this bacterium, as well as previous outbreak reports that identified the environment as source/reservoir [13, 17].

The case-control studies showed first that previous use of certain antibiotics were associated with an increased risk of acquisition of VIM-PA; especially the use of quinolones, piperacillin/tazobactam, and trimethoprim/sulfamethoxazole should be avoided if possible. Second, gastroscopy and bronchoscopy were identified as risk factors (Fig. 2). Third, the results of the two different case-control studies were largely in line with each other, with three common risk factors (i.e. previous use of quinolones, use of SDD for > 10 days, and having undergone ≥1 gastroscopy 6 months prior to the identification of VIM-PA) that could therefore be considered as robust. The assumption would be that certain antibiotics change the normal gut or throat flora in such a way that multidrug-resistant bacteria more easily attach to and colonize either the gut or throat. Nevertheless, multidrug-resistant microorganisms have to be offered to the patient, and this may occur through endoscopic procedures by contaminated endoscopes or using water from a contaminated source. Both the previous use of antibiotics and prior procedures with flexible endoscopes have been highlighted in previous studies as risk factors for acquisition of various multidrug microorganisms, including VIM-PA [18, 19].

The group of antibiotics that favours presence of VIM-PA (i.e. increases a patients’ susceptibility to acquire VIM-PA) depends on the choice of the control group. Furthermore, we learned that although highly significant factors were obtained with one group of controls, these can disappear when other groups are compared as these groups differ in inclusion criteria or definition; the results highly depend on the choice of the control.

### Limitations

#### Network analysis

Criteria for epidemiological relationships, especially relationships in time and space, are not clearly defined for outbreaks with multidrug-resistant bacteria. We developed criteria which are easy to apply; however, inherent to this is a simplification of the truth. We propose that these definitions would be modified or extended in case data from future studies warrants.

#### Case-control studies

First, this is a single-centre case-control study, which possibly hampers generalizability. Second, matching on ward of acquisition and length of stay prior to the positive culture might have caused additional matching on e.g. comorbidities and disease severity. However, we have done this deliberately. When comparing ICU to non-ICU patients, disease severity and possibly also comorbidities will be risk factors just because the groups are not similar. Third, misclassification of exposure could be present; not all control patients were cultured for VIM-PA, which could lead to VIM-PA carriers present in control group. However, this misclassification if present could only have led to an underestimation of the identified effects. Fourth, perfect matching was only achieved in 38%. This seems low; however, 100% perfect matching is not possible for large case-control studies including patients who have complicated medical histories and futures. Possibly, the percentage of perfect matching could be added as an item to the STROBE statement [20].

In one of the subgroup analyses, differences were identified between DiversiLab clonal cluster A and B (Fig. 3). However, although widely applied, the DiversiLab system can be considered a limitation of the study, since available data regarding the DiversiLab system for *P. aeruginosa* are contradictory. A review by Brossier et al. on the performance of the DiversiLab system for *P. aeruginosa* concluded that the results should be interpreted with caution, and always in combination with epidemiological data, as was done in our study [21].

## Conclusion

The network analysis indicated that the majority of transmissions occurred on the wards, but through unidentified and presumably persistent sources, which are most likely in the innate hospital environment. Previous use of certain antibiotic regimens made patients prone to VIM-PA carriage. Additionally, gastroscopy could be considered as a high-risk procedure in patients with risk factors.

### Recommendation

If there is an outbreak with VIM-PA, we showed that first; the entire ward should be seen as reservoir and as contaminated. Therefore, cleaning and disinfection practices should be installed and possible sources should be eliminated. We also feel that it is especially important to search for unknown reservoirs in the environment. Second, use of particularly quinolones should be avoided because this could make a patient ‘prone’ for acquiring VIM-PA. Third, we showed that in an outbreak setting gastroscopy and bronchoscopy could be seen as high-risk procedures. Finally, a case-control study should be executed to identify outbreak specific risk factors. Because we showed that if you change matching criteria outcomes do differ, it would be advisable to always include multiple definitions for control inclusion.

## Additional files

Additional file 1: Text file: The number of clinical admissions and clinical admission days and the number of patients included in this study from 2003 until 2015. (DOCX 19 kb)

Additional file 2: Text file: List of all variables extracted from electronic medical records of included case and control patients. (DOCX 28 kb)

Additional file 3: Text file: Treatment related variables, categorical, univariate analysis. Variables with an asterisk (*) were selected for multivariable analysis. (DOCX 37 kb)

Additional file 4: Text file: Subgroup analysis DiversiLab (bioMérieux) type A and type B, univariate analysis. Variables with an asterisk (*) were selected for multivariable analysis. (DOCX 70 kb)

Additional file 5: Text file: Subgroup analysis ICU or non-ICU as ward of acquisition of VIM-PA, univariate analysis. Variables with an asterisk (*) were selected for multivariable analysis. (DOCX 83 kb)

Additional file 6: Text file: Crude odds ratios; control group 1&2 versus control group 3&4. Variables with an asterisk (*) were selected for multivariable analysis. (DOCX 35 kb)

## Abbreviations

CI: Confidence interval
Erasmus MC: Erasmus MC University Medical Centre
ICUs: Intensive care units
MBL: Metallo-β-lactamases
MDRPA: Multidrug-resistant strains of *Pseudomonas aeruginosa*
OR: Odds ratio
PCR: Polymerase chain reaction
SDD: Selective digestive tract decontamination
VIM: Verona Integron-encoded metallo-β-lactamase
VIM-PA: Verona Integron-encoded metallo-β-lactamase-positive clone of *Pseudomonas aeruginosa*
