# Supplementary material for: VIM-positive Pseudomonas aeruginosa in a large tertiary care hospital: matched case-control studies and a network analysis
Source: Antimicrob Resist Infect Control. 2018 Feb 27;7:32. doi: 10.1186/s13756-018-0325-1 (PMC5828133; doi:10.1186/s13756-018-0325-1)
Supplement: Supplementary file 1 — Text file: The number of clinical admissions and clinical admission days and the number of patients included in this study from 2003 until 2015. (DOCX 19 kb) [file 13756_2018_325_MOESM1_ESM.docx]

**Additional file 1: Text file:** The number of clinical admissions and clinical admission days and the number of patients included in this study from 2003 until 2015.

| Year | Number of clinical admissions | Number of clinical admission days | Number of patients included in this study^1^ |
| --- | --- | --- | --- |
| 2003 | 34,568 | 316,157 | 1 |
| 2004 | 35,985 | 314,295 | 0 |
| 2005 | 36,001 | 315,475 | 3 |
| 2006 | 36,818 | 310,256 | 2 |
| 2007 | 37,044 | 309,891 | 2 |
| 2008 | 38,141 | 311,114 | 10 |
| 2009 | 38,865 | 306,860 | 25 |
| 2010 | 40,443 | 311,873 | 26 |
| 2011 | 41,497 | 304,219 | 34 |
| 2012 | 41,001 | 300,947 | 18 |
| 2013 | 37,134 | 293,598 | 11 |
| 2014 | 36,976 | 288,865 | 10 |
| 2015 | 36,853 | 289,018 | 2 |

^1^Patients identified with Verona Integron-encoded Metallo-β-lactamase (VIM)-positive *Pseudomonas aeruginosa*.
