# Supplementary material for: VIM-positive Pseudomonas aeruginosa in a large tertiary care hospital: matched case-control studies and a network analysis
Source: Antimicrob Resist Infect Control. 2018 Feb 27;7:32. doi: 10.1186/s13756-018-0325-1 (PMC5828133; doi:10.1186/s13756-018-0325-1)
Supplement: Supplementary file 2 — Text file: List of all variables extracted from electronic medical records of included case and control patients. (DOCX 28 kb) [file 13756_2018_325_MOESM2_ESM.docx]

**Additional file 2: Text file:** List of all variables extracted from electronic medical records of included case and control patients.

| **Variable** | **Explanation** |
| --- | --- |
| Patient characteristics |  |
| Patient identification number | NA |
| Date of birth; age | Case: age at time of identification VIM-PA  Control: age at time of detection VIM-PA in case |
| Gender | Male/female |
| Ward of acquisition | Case: ward of acquisition: ward where patient was admitted 48h before identification of VIM-PA.  Control: matched; control must be admitted at the same ward as ward of acquisition of case. |
| Date of death | Case: within 28 days after identification of VIM-PA, 1y after identification of VIM-PA  Control: within 28 days after discharge, 1y after discharge |
| Date of admittance Erasmus MC | Case: admission during which the VIM-PA was identified.  Control: admission when matched to case patient. |
| Transferred from another hospital | Yes/no |
| Date of discharge Erasmus MC | NA |
| Date of admittance ward of acquisition | NA |
| Date of discharge ward of acquisition | NA |
| Being admitted at ICU 1y before VIM-PA | Yes/no, control: using the VIM-PA date of case |
| Being admitted in Erasmus MC 1y before VIM-PA | Yes/no, control: using the VIM-PA date of case |
| Cystic fibrosis | Yes/no |
| Chronic respiratory illness | Yes/no, COPD, asthma, CF, lung fibrosis, lung malignancies, tuberculosis. |
| Acute respiratory illness | Yes/no; needing mechanical ventilation |
| Chronic kidney failure | Yes/no; dependent on intermittent hemodialysis or continuous ambulatory peritoneal dialysis. |
| Acute kidney failure | Yes/no; dependent on continuous veno-venous hemofiltration or intermittent hemodialysis or continuous ambulatory peritoneal dialysis started during admission. |
| Chronic liver failure | Yes/no; being on the liver transplantation (LTx ) waiting list |
| Acute liver failure | Yes/no; being on the highly urgent liver transplantation (HULTx) waiting list |
| Chronic problems of the gastrointestinal tract | Yes/no; *e.g.* malignancies, irritable bowel syndrome, inflammatory bowel disease. |
| Acute problems of the gastrointestinal tract | Yes/no; *e.g.* gastroenteritis, gastrointestinal abscesses, ileus, ischemia, intestinal torsion (volvulus), fistula. |
| Auto-immune disease | Yes/no; all known auto-immune diseases |
| Human immunodeficiency virus | Yes/no; patients with positive HIV serology |
| Diabetes | Yes/no; yype I or type II diabetes |
| Solid organ transplant recipient | Yes/no, year of transplant |
| Stem cell or bone marrow transplant | Yes/no, year of transplant |
| Immunocompromised | Yes/no; use of immunosuppressants, HIV, neutropenia, transplant recipient. |
| Neutropenia | Yes/no; neutrophil count <500µL |
| Surgery | Yes/no; number of surgeries from admission to identification of VIM-PA. |
| Medical devices | Case: from admission until date of identification of VIM-PA.  Control: from admission until the VIM-PA identification date of case |
| Mechanical ventilation | Yes/no, only possible when admitted at ICU |
| Tracheostomy | Yes/no |
| Extracorporeal membrane oxygenation | Yes/no, only possible when admitted at ICU |
| Central venous catheter | Yes/no |
| Urinary catheter | Yes/no |
| Drains | Yes/no |
| Endoscopies | Case: 6 months before identification of VIM-PA  Control: Similar time window (in days) as the case patient  Categories: 1) yes/no, 2) number |
| Colonoscopy | NA |
| Sigmoidoscopy | NA |
| Endoscopic ultrasound | NA |
| Gastroscopy | Diagnostic or for placement of a feeding tube/ duodenal tube. |
| ERCP | NA |
| Bronchoscopy | NA |
| Transesophageal Echocardiography (TEE) | NA |
| Prior use of: | Case: 6 months before identification of VIM-PA  Control: Similar time window (in days) as the case patient  Categories: 1) use yes/no, 2) use for 0/1-3/≥4 days or 3) use for 0/1-3/4-10/≥11 days |
| Antifungals | All possible antifungal drugs |
| Antivirals | All possible antiviral drugs |
| Aminoglycosides | NA |
| Amoxicillin/clavulanic acid | Also known as Augmentin |
| Carbapenems | Imipenem and/or meropenem |
| Cephalosporins | NA |
| Colistin | NA |
| Macrolides | NA |
| Metronidazole | NA |
| Nitrofurantoin | NA |
| Penicillin | NA |
| Piperacillin/tazobactam | NA |
| Quinolones | NA |
| Trimethoprim/sulfamethoxazole | Also known as co-trimoxazole |
| Vancomycin | NA |
| Other antibiotics | NA |
| Selective digestive tract decontamination (SDD) | If a patients is expected to be on a mechanical ventilator for >48h or is expected to be admitted to the ICU for >3 days. For SDD regimen: de Smet et al. 2009 [1]; in addition, at the Erasmus MC we use the SDD regimen with cefotaxime. |

Abbreviations: VIM-PA= Verona Integron-encoded Metallo-β-lactamase (VIM)-positive *Pseudomonas aeruginosa*, ICU= intensive care unit, HIV= human immunodeficiency virus, COPD= chronic obstructive pulmonary disease, CF= cystic fibrosis, y= year, h= hour(s), NA= not applicable.

1. de Smet AM, Kluytmans JA, Cooper BS, et al. Decontamination of the digestive tract and oropharynx in ICU patients. N Engl J Med **2009**; 360(1): 20-31.
