# Supplementary material for: VIM-positive Pseudomonas aeruginosa in a large tertiary care hospital: matched case-control studies and a network analysis
Source: Antimicrob Resist Infect Control. 2018 Feb 27;7:32. doi: 10.1186/s13756-018-0325-1 (PMC5828133; doi:10.1186/s13756-018-0325-1)
Supplement: Supplementary file 3 — Text file: Treatment related variables, categorical, univariate analysis. Variables with an asterisk (*) were selected for multivariable analysis. (DOCX 37 kb) [file 13756_2018_325_MOESM3_ESM.docx]

**Additional file 3.** Treatment related variables, categorical, univariate analysis.

1. Categories: 0 days, 1-3 days, 4-10 days and ≥11 days

| Variables | Cases  (n= 138) | Controls 1&2 (n= 288) | Crude OR  (95% CI), P-value | Controls 3&4 (n= 288) | Crude OR  (95% CI), P-value |
| --- | --- | --- | --- | --- | --- |
| Aminoglycosides (%) |  |  |  |  |  |
| 0 days | 87 (63.0) | 235 (81.6)* | Reference | 264 (91.7) | Reference |
| 1-3 days | 29 (21.0) | 24 (8.3)* | **3.878 (1.987-7.568), <0.001** | 7 (2.4) | **12.633 (4.796-33.275), <0.001** |
| 4-10 days | 10 (7.2) | 18 (6.3)* | **1.629 (0.663-4.003), 0.288** | 9 (3.1) | **4.560 (1.505-13.811), 0.007** |
| ≥11 days | 12 (8.7) | 11 (3.8)* | **3.222 (1.334-7.783), 0.009** | 8 (2.8) | **3.713 (1.460-9.440), 0.006** |
| Amoxicillin/clavulanic acid (%) |  |  |  |  |  |
| 0 days | 99 (71.7) | 240 (83.3) | Reference | 248 (86.1) | Reference |
| 1-3 days | 15 (10.9) | 18 (6.3) | 2.045 (0.953-4.389), 0.066 | 18 (6.3) | 2.018 (0.978-4.163), 0.058 |
| 4-10 days | 22 (15.9) | 29 (10.1) | 1.887 (0.995-3.577), 0.052 | 19 (6.6) | **2.845 (1.450-5.581), 0.002** |
| ≥11 days | 2 (1.4) | 1 (0.3) | 4.542 (0.407-40.743), 0.219 | 3 (1.0) | 1.747 (0.281-10.859), 0.550 |
| Carbapenems (%) |  |  |  |  |  |
| 0 days | 80 (58.0) | 230 (79.9)* | Reference | 262 (91.0) | Reference |
| 1-3 days | 10 (7.2) | 16 (5.6)* | 1.854 (0.775-4.435), 0.165 | 9 (3.1) | **2.795 (1.060-7.371), 0.038** |
| 4-10 days | 24 (17.4) | 26 (9.0)* | **3.001 (1.551-5.808), 0.001** | 12 (4.2) | **7.540 (3.163-17.979), <0.001** |
| ≥11 days | 24 (17.4) | 16 (5.6)* | **4.556 (2.209-9.398), <0.001** | 5 (1.7) | **13.522 (4.864-37.591), <0.001** |
| Cephalosporins (%) |  |  |  |  |  |
| 0 days | 34 (24.6) | 157 (54.5)* | Reference | 181 (62.8) | Reference |
| 1-3 days | 32 (23.2) | 41 (14.2)* | **3.986 (2.026-7.844), <0.001** | 51 (17.7) | **4.908 (2.484-9.697), <0.001** |
| 4-10 days | 45 (32.6) | 70 (24.3)* | **3.193 (1.797-5.673), <0.001** | 46 (16.0) | **5.690 (3.002-10.786), <0.001** |
| ≥11 days | 27 (19.6) | 20 (6.9)* | **6.928 (3.219-14.910), <0.001** | 10 (3.5) | **23.535 (8.376-66.130), <0.001** |
| Colistin (%) |  |  |  |  |  |
| 0 days | 121 (87.7) | 268 (93.1) | Reference | 276 (95.8) | Reference |
| 1-3 days | 3 (2.2) | 4 (1.4) | 1.575 (0.349-7.113), 0.555 | 1 (0.3) | 6.000 (0.624-57.681), 0.121 |
| 4-10 days | 6 (4.3) | 9 (3.1) | 1.378 (0.487-3.900), 0.546 | 4 (1.4) | **5.966 (1.159-3..715), 0.033** |
| ≥11 days | 8 (5.8) | 7 (2.4) | 3.176 (0.917-11.002), 0.068 | 7 (2.4) | **3.256 (1.030-10.291), 0.044** |
| Macrolides (%) |  |  |  |  |  |
| 0 days | 78 (56.5) | 224 (77.8)* | Reference | 261 (90.6) | Reference |
| 1-3 days | 20 (14.5) | 30 (10.4)* | 1.805 (0.934-3.489), 0.079 | 11 (3.8) | **5.254 (2.379-11.604), <0.001** |
| 4-10 days | 29 (21.0) | 28 (9.7)* | **3.236 (1.693-6.183), <0.001** | 15 (5.2) | **6.418 (3.069-13.419), <0.001** |
| ≥11 days | 11 (8.0) | 6 (2.1)* | **4.734 (1.702-13.165), 0.003** | 1 (0.3) | **34.521 (4.245-280.721), 0.001** |
| Metronidazole (%) |  |  |  |  |  |
| 0 days | 90 (65.2) | 240 (83.3)* | Reference | 256 (88.9) | Reference |
| 1-3 days | 15 (10.9) | 14 (4.9)* | **2.750 (1.257-6.013), 0.011** | 12 (4.2) | **5.266 (1.988-13.945), 0.001** |
| 4-10 days | 15 (10.9) | 25 (8.7)* | 1.405 (0.718-2.751), 0.321 | 18 (6.3) | **2.175 (1.002-4.721), 0.049** |
| ≥11 days | 18 (13.0) | 9 (3.1)* | **6.105 (2.358-15.804), <0.001** | 2 (0.7) | **54.608 (6.848-435.455), <0.001** |
| Penicillin (%) |  |  |  |  |  |
| 0 days | 109 (79.0) | 239 (83.0) | Reference | 257 (89.2) | Reference |
| 1-3 days | 9 (6.5) | 12 (4.2) | 1.524 (0.625-3.717), 0.354 | 14 (4.9) | 1.471 (0.627-3.450), 0.375 |
| 4-10 days | 11 (8.0) | 25 (8.7) | 0.923 (0.444-1.920), 0.830 | 14 (4.9) | 1.775 (0.765-4.119), 0.182 |
| ≥11 days | 9 (6.5) | 12 (4.2) | 1.556 (0.618-3.913), 0.348 | 3 (1.0) | **6.407 (1.724-23.809), 0.006** |
| Piperacillin/tazobactam (%) |  |  |  |  |  |
| 0 days | 89 (64.5) | 238 (82.6) | Reference | 256 (88.9) | Reference |
| 1-3 days | 22 (15.9) | 22 (7.6) | **2.847 (1.404-5.773), 0.004** | 15 (5.2) | **4.492 (2.049-9.848), <0.001** |
| 4-10 days | 22 (15.9) | 20 (6.9) | **3.012 (1.492-6.080), 0.002** | 13 (4.5) | **4.985 (2.231-11.138), <0.001** |
| ≥11 days | 5 (3.6) | 8 (2.8) | 1.870 (0.587-5.960), 0.290 | 4 (1.4) | 3.558 (0.908-13.938), 0.069 |
| Quinolones (%) |  |  |  |  |  |
| 0 days | 44 (31.9) | 200 (69.4)* | Reference | 235 (81.6) | Reference |
| 1-3 days | 18 (13.0) | 30 (10.4)* | **3.428 (1.598-7.357), 0.002** | 16 (5.6) | **5.978 (2.627-13.603), <0.001** |
| 4-10 days | 43 (31.2) | 37 (12.8)* | **6.710 (3.446-13.069), <0.001** | 20 (6.9) | **10.359 (5.143-20.864), <0.001** |
| ≥11 days | 33 (23.9) | 21 (7.3)* | **8.753 (4.161-18.411), <0.001** | 17 (5.9) | **10.839 (4.911-23.925), <0.001** |
| Trimethoprim/sulfamethoxazole (%) |  |  |  |  |  |
| 0 days | 106 (76.8) | 265 (92.0) | Reference | 269 (93.4) | Reference |
| 1-3 days | 7 (5.1) | 4 (1.4) | **4.637 (1.299-16.550), 0.018** | 2 (0.7) | **7.000 (1.454-33.696), 0.015** |
| 4-10 days | 11 (8.0) | 8 (2.8) | **3.968 (1.397-11.272), 0.010** | 4 (1.4) | **5.739 (1.821-18.082), 0.003** |
| ≥11 days | 14 (10.1) | 11 (3.8) | **3.088 (1.337-7.129), 0.008** | 13 (4.5) | **2.550 (1.145-5.678), 0.022** |
| Vancomycin (%) |  |  |  |  |  |
| 0 days | 64 (46.4) | 237 (82.3) | Reference | 261 (90.6) | Reference |
| 1-3 days | 21 (15.2) | 16 (5.6) | **4.003 (1.958-8.182), <0.001** | 11 (3.8) | **5.975 (2.626-13.599), <0.001** |
| 4-10 days | 23 (16.7) | 26 (9.0) | **4.673 (2.140-10.208), <0.001** | 11 (3.8) | **8.903 (3.677-13.599), <0.001** |
| ≥11 days | 30 (21.7) | 9 (3.1) | **27.311 (7.799-95.639), <0.001** | 5 (1.7) | **20.932 (6.971-62.848), <0.001** |
| Selective digestive tract decontamination (%) |  |  |  |  |  |
| 0 days | 46 (33.3) | 179 (62.2)* | Reference | 229 (79.5)* | Reference |
| 1-3 days | 17 (12.3) | 20 (6.9)* | **3.349 (1.476-7.602), 0.004** | 18 (6.3)* | **5.021 (1.527-16.502), 0.008** |
| 4-10 days | 20 (14.5) | 47 (16.3)* | 1.928 (0.886-4.194), 0.098 | 25 (8.7)* | 1.963 (0.876-4.402), 0.102 |
| ≥11 days | 55 (39.9) | 42 (14.6)* | **10.202 (4.681-22.232), <0.001** | 16 (5.6)* | **2.749 (1.023-7.385), 0.045** |

1. Categories: 0 days, 1-3 days, ≥4 days

| Variables | Cases  (n= 138) | Controls 1&2 (n= 288) | Crude OR  (95% CI), P-value | Controls 3&4 (n= 288) | Crude OR  (95% CI), P-value |
| --- | --- | --- | --- | --- | --- |
| Aminoglycosides (%) |  |  |  |  |  |
| 0 days | 87 (63.0) | 235 (81.6) | Reference | 264 (91.7)* | Reference |
| 1-3 days | 29 (21.1) | 24 (8.3) | **3.863 (1.981-7.533), <0.001** | 7 (2.4)* | **12.480 (4.766-32.681), <0.001** |
| ≥4 days | 22 (15.9) | 29 (10.1) | **2.305 (1.191-4.459), 0.013** | 17 (5.9)* | **4.030 (1.844-8.806), <0.001** |
| Amoxicillin/clavulanic acid (%) |  |  |  |  |  |
| 0 days | 99 (71.7) | 240 (83.3) | Reference | 248 (86.1) | Reference |
| 1-3 days | 15 (10.9) | 18 (6.3) | 2.066 (0.962-4.436), 0.063 | 18 (6.3) | 2.022 (0.980-4.172), 0.057 |
| ≥4 days | 24 (17.4) | 30 (10.4) | **1.983 (1.063-3.700), 0.031** | 22 (7.6) | **2.705 (1.423-5.142), 0.002** |
| Carbapenems (%) |  |  |  |  |  |
| 0 days | 80 (58.0) | 230 (79.9) | Reference | 262 (91.0)* | Reference |
| 1-3 days | 10 (7.2) | 16 (5.6) | 1.902 (0.797-4.540), 0.147 | 9 (3.1)* | **2.793 (1.060-7.359), 0.038** |
| ≥4 days | 48 (34.8) | 42 (14.6) | **3.619 (2.095-6.250), <0.001** | 17 (5.9)* | **9.780 (4.764-20.079), <0.001** |
| Cephalosporins (%) |  |  |  |  |  |
| 0 days | 34 (24.6) | 157 (54.5) | Reference | 181 (62.8)* | Reference |
| 1-3 days | 32 (23.2) | 41 (14.2) | **3.929 (2.015-7.661), <0.001** | 51 (17.7)* | **4.279 (2.218-8.254), <0.001** |
| ≥4 days | 72 (52.2) | 90 (31.3) | **3.947 (2.307-6.752), <0.001** | 56 (19.4)* | **8.171 (4.469-14.939), <0.001** |
| Colistin (%) |  |  |  |  |  |
| 0 days | 121 (87.7) | 268 (93.1) | Reference | 276 (95.8) | Reference |
| 1-3 days | 3 (2.2) | 4 (1.4) | 1.672 (0.370-7.561), 0.504 | 1 (0.3) | 6.000 (0.624-57.681), 0.121 |
| ≥4 days | 14 (10.1) | 16 (5.6) | 1.952 (0.896-4.250), 0.092 | 11 (3.8) | **4.000 (1.515-10-563), 0.005** |
| Macrolides (%) |  |  |  |  |  |
| 0 days | 78 (56.5) | 224 (77.8) | Reference | 261 (90.6)* | Reference |
| 1-3 days | 20 (14.5) | 30 (10.4) | 1.828 (0.946-3.532), 0.073 | 11 (3.8)* | **5.311 (2.408-11.715), <0.001** |
| ≥4 days | 40 (29.0) | 34 (11.8) | **3.570 (1.992-6.397), <0.001** | 16 (5.6)* | **8.179 (4.058-16.483), <0.001** |
| Metronidazole (%) |  |  |  |  |  |
| 0 days | 90 (65.2) | 240 (83.3) | Reference | 256 (88.9) | Reference |
| 1-3 days | 15 (10.9) | 14 (4.9) | **2.680 (1.230-5.838), 0.013** | 12 (4.2) | **4.102 (1.724-9.758), 0.001** |
| ≥4 days | 33 (23.9) | 34 (11.8) | **2.355 (1.383-4.010), 0.002** | 20 (6.9) | **4.997 (2.584-9.666), <0.001** |
| Penicillin (%) |  |  |  |  |  |
| 0 days | 109 (79.0) | 239 (83.0) | Reference | 257 (89.2)* | Reference |
| 1-3 days | 9 (6.5) | 12 (4.2) | 1.546 (0.634-3.770), 0.338 | 14 (4.9)* | 1.455 (0.623-3.399), 0.385 |
| ≥4 days | 20 (14.5) | 37 (12.8) | 1.113 (0.614-2.019), 0.724 | 17 (5.9)* | **2.671 (1.331-5.362), 0.006** |
| Piperacillin/tazobactam (%) |  |  |  |  |  |
| 0 days | 89 (64.5) | 238 (82.6) | Reference | 256 (88.9) | Reference |
| 1-3 days | 22 (15.9) | 22 (7.6) | **2.901 (1.434-5.878), 0.003** | 15 (5.2) | **4.530 (2.065-9.937), <0.001** |
| ≥4 days | 27 (19.6) | 28 (9.7) | **2.683 (1.434-5.020), 0.002** | 17 (31.3) | **4.604 (2.266-9.356), <0.001** |
| Quinolones (%) |  |  |  |  |  |
| 0 days | 44 (31.9) | 200 (69.4) | Reference | 235 (81.6)* | Reference |
| 1-3 days | 18 (13.0) | 30 (10.4) | **3.365 (1.573-7.197), 0.002** | 16 (5.6)* | **5.972 (2.626-13.585), <0.001** |
| ≥4 days | 76 (55.1) | 58 (20.1) | **7.512 (4.184-13.488), <0.001** | 37 (12.8)* | **10.555 (5.799-19.212), <0.001** |
| Trimethoprim/sulfamethoxazole (%) |  |  |  |  |  |
| 0 days | 106 (76.8) | 265 (92.0) | Reference | 269 (93.4) | Reference |
| 1-3 days | 7 (5.1) | 4 (1.4) | **4.599 (1.292-16.364), 0.018** | 2 (0.7) | **7.000 (1.454-33.696), 0.015** |
| ≥4 days | 25 (18.1) | 19 (6.6) | **3.405 (1.735-6.681), <0.001** | 17 (5.9) | **3.385 (1.752-6.538), <0.001** |
| Vancomycin (%) |  |  |  |  |  |
| 0 days | 64 (46.4) | 237 (82.3)* | Reference | 261 (90.6)* | Reference |
| 1-3 days | 21 (15.2) | 16 (5.6)* | **4.438 (2.152-9.156), <0.001** | 11 (3.8)* | **6.285 (2.736-14.435), <0.001** |
| ≥4 days | 53 (38.4) | 35 (12.2)* | **8.296 (4.193-16.413), <0.001** | 16 (5.6)* | **12.907 (6.179-26.961), <0.001** |
| Selective digestive tract decontamination (%) |  |  |  |  |  |
| 0 days | 46 (33.3) | 179 (62.2) | Reference | 229 (79.5) | Reference |
| 1-3 days | 17 (12.3) | 20 (6.9) | **3.864 (1.714-8.711), 0.001** | 18 (6.3) | **7.899 (3.159-19.750), <0.001** |
| ≥4 days | 75 (54.3) | 89 (30.9) | **4.871 (2.685-8.836), <0.001** | 41 (14.2) | **11.709 (6.055-22.642), <0.001** |

Abbreviations: 95% CI= 95% confidence interval, OR= odds ratio, bold= statistically significant (p<0.05), *= included in the multivariable analysis

For nitrofurantoin, no antibiotic days were available, so this antibiotic could only be included as a yes/no variable. For antiviral, antifungal and other antibiotic use we decided to also only use the yes/no variable.
