# Supplementary material for: VIM-positive Pseudomonas aeruginosa in a large tertiary care hospital: matched case-control studies and a network analysis
Source: Antimicrob Resist Infect Control. 2018 Feb 27;7:32. doi: 10.1186/s13756-018-0325-1 (PMC5828133; doi:10.1186/s13756-018-0325-1)
Supplement: Supplementary file 4 — Text file: Subgroup analysis DiversiLab (bioMérieux) type A and type B, univariate analysis. Variables with an asterisk (*) were selected for multivariable analysis. (DOCX 70 kb) [file 13756_2018_325_MOESM4_ESM.docx]

**Additional file 4: Text file:** Subgroup analysis DiversiLab (bioMérieux) clonal cluster A and clonal cluster B, univariate analysis.

**Table 1a.** Patient related clinical variables of case patients with DiversiLab (bioMérieux) clonal cluster A, univariate analysis

| Variables | Cases  (n= 29) | Controls 1&2 (n= 58) | Crude OR  (95% CI), *P*-value | Controls 3&4 (n= 58) | Crude OR  (95% CI), *P*-value |
| --- | --- | --- | --- | --- | --- |
| Basic characteristics |  |  |  |  |  |
| Age, years, median (range) | 55.5 (27-81) | 60.5 (19-92) | 0.986 (0.956-1.016), 0.357 | 63.5 (25-91)* | **0.964 (0.930-0.998), 0.040** |
| Male gender (%) | 15 (51.7) | 32 (55.2) | 0.854 (0.329-2.218), 0.746 | 31 (53.4) | 0.934 (0.384-2.271), 0.880 |
| 28-day mortality (%) | 6 (20.7) | 16 (27.6) | 0.681 (0.234-1.984), 0.481 | 8 (13.8) | 1.618 (0.505-5.185), 0.418 |
| 1-year mortality (%) | 12 (41.4) | 21 (36.2) | 1.290 (0.475-3.504), 0.618 | 15 (25.9) | 2.306 (0.805-6.606), 0.120 |
| Transferred from another hospital (%) | 11 (37.9) | 13 (22.4) | 1.916 (0.772-4.752), 0.161 | 11 (19.0)* | 2.288 (0.901-5.811), 0.082 |
| Median length of admission (range) | 61 (7-243) | 27 (1-172) | **1.020 (1.006-1.035), 0.006** | 12 (1-302) | **1.024 (1.009-1.040), 0.001** |
| Erasmus MC 1y before VIM-PA (%) | 6 (20.7) | 25 (43.1) | 0.377 (0.135-10.54), 0.063 | 22 (37.9)* | 0.355 (0.109-1.161), 0.087 |
| Erasmus MC ICU 1y before VIM-PA (%) | 2 (6.9) | 3 (5.2) | NA | 2 (3.4) | NA |
| Surgery (%) | 21 (72.4) | 31 (53.4)* | **2.042 (0.835-4.994), 0.118** | 24 (41.4)* | **3.758 (1.340-10.536), 0.012** |
| Underlying diseases |  |  |  |  |  |
| Cystic fibrosis (%) | 0 (0) | 0 (0) | NA | 0 (0) | NA |
| Chronic respiratory illness (%) | 6 (20.7) | 7 (12.1) | 1.804 (0.572-5.689), 0.314 | 9 (15.5) | 1.429 (0.453-4.501), 0.542 |
| Chronic kidney failure (%) | 1 (3.4) | 1 (1.7) | NA | 2 (3.4) | NA |
| Acute kidney failure; use of CVVH (%) | 4 (13.8) | 7 (12.1) | NA | 4 (6.9) | NA |
| Chronic liver failure (%) | 0 (0) | 0 (0) | NA | 1 (1.7) | NA |
| Acute liver failure (%) | 0 (0) | 1 (1.7) | NA | 0 (0) | NA |
| Chronic problems of the gastrointestinal tract (%) | 3 (10.3) | 5 (8.6) | NA | 3 (5.2) | NA |
| Acute problems of the gastrointestinal tract (%) | 7 (24.1) | 12 (20.7) | 1.189 (0.442-3.197), 0.732 | 12 (20.7) | 1.202 (0.432-3.345), 0.724 |
| Auto-immune disease (%) | 2 (6.9) | 3 (5.2) | NA | 5 (8.6) | NA |
| Human immunodeficiency virus (%) | 0 (0) | 2 (3.4) | NA | 0 (0) | NA |
| Diabetes (%) | 3 (10.3) | 5 (8.6) | NA | 6 (10.3) | NA |
| Solid organ transplant recipient (%) | 2 (6.9) | 3 (5.2) | NA | 5 (8.6) | NA |
| Stem cell/bone marrow transplant recipient (%) | 1 (3.4) | 1 (1.7) | NA | 0 (0) | NA |
| Use of immunosuppressive agents (%) | 12 (41.4) | 17 (29.3) | 1.769 (0.672-4.659), 0.248 | 8 (13.8)* | **4.000 (1.383-11.566), 0.010** |
| Malignancies (%) | 4 (13.8) | 15 (25.9) | NA | 18 (31.0) | NA |
| Neutropenia, <500 cells/µL (%) | 1* (4.5) | 0** (0) | NA | 1*** (3.1) | NA |
| Endoscopies |  |  |  |  |  |
| Colonoscopy (%) | 1 (3.4) | 2 (3.4) | NA | 2 (3.4) | NA |
| Sigmoidoscopy (%) | 0 (0) | 2 (3.4) | NA | 3 (5.2) | NA |
| Endoscopic ultrasound (%) | 1 (3.4) | 3 (5.2) | NA | 6 (10.3) | NA |
| Gastroscopy (%) | 16 (55.2) | 12 (20.7)* | **5.439 (1.760-16.809), 0.003** | 14 (24.1)* | **4.784 (1.535-14.911), 0.007** |
| ERCP (%) | 3 (10.3) | 3 (5.2) | NA | 1 (1.7) | NA |
| Bronchoscopy (%) | 5 (17.2) | 9 (15.5) | 1.131 (0.349-3.703), 0.838 | 5 (8.6) | 2.195 (0.576-8.362), 0.249 |
| Transesophageal Echocardiography (TEE) (%) | 3 (10.3) | 3 (5.2) | NA | 5 (8.6) | NA |
| Medical devices |  |  |  |  |  |
| Mechanical ventilation (%) | 29 (100) | 44 (75.9) | NA | 32 (55.2) | NA |
| Tracheostomy (%) | 6 (20.7) | 8 (13.8) | 1.667 (0.509-5.461), 0.399 | 2 (3.4) | NA |
| Extracorporeal membrane oxygenation (%) | 0 (0) | 0 (0) | NA | 0 (0) | NA |
| Central venous catheter (%) | 20 (69.0) | 22 (37.9)* | **3.386 (1.293-8.865), 0.013** | 19 (32.8)* | **5.411 (1.765-16.585), 0.003** |

Abbreviations: VIM-PA= Verona Integron-encoded Metallo-β-lactamase (VIM)-positive *Pseudomonas aeruginosa*, 95% CI= 95% confidence interval, OR= odds ratio, y= year, ICU= intensive care unit, CVVH= Continuous Veno-Venous Hemofiltration, ERCP= Endoscopic Retrograde Cholangiopancreatography, NA= not applicable. *for 7 patients information was missing, **for 17 patients information was missing, ***for 26 patients information was missing.**Table 1b.** Treatment related variables of case patients with DiversiLab (bioMérieux) clonal cluster A, univariate analysis

| Variables | Cases  (n= 29) | Controls 1&2 (n= 58) | Crude OR  (95% CI), *P*-value | Controls 3&4 (n= 58) | Crude OR  (95% CI), *P*-value |
| --- | --- | --- | --- | --- | --- |
| Treatment related variables; yes/no |  |  |  |  |  |
| Antifungals (%) | 16 (55.2) | 15 (25.9)* | **7.663 (1.679-34.980), 0.009** | 8 (13.8)* | **8.309 (2.379-29.012), 0.001** |
| Antivirals (%) | 3 (10.3) | 1 (1.7) | NA | 3 (5.2) | NA |
| Aminoglycosides (%) | 10 (34.5) | 8 (13.8)* | **3.221 (1.074-9.659), 0.037** | 7 (12.1)* | **3.475 (1.169-10.336), 0.025** |
| Amoxicillin/clavulanic acid (%) | 5 (17.2) | 12 (20.7) | 0.772 (0.223-2.677), 0.684 | 9 (15.5) | 1.131 (0.346-3.703), 0.838 |
| Carbapenems (%) | 15 (51.7) | 13 (22.4)* | **3.874 (1.360-11.038), 0.011** | 4 (6.9) | NA |
| Cephalosporins (%) | 23 (79.3) | 23 (39.7) | **5.355 (1.775-16.154), 0.003** | 20 (34.5) | **8.690 (2.509-30.099), 0.001** |
| Colistin (%) | 4 (13.8) | 7 (12.1) | NA | 1 (1.7) | NA |
| Macrolides (%) | 18 (62.1) | 17 (29.3)* | **3.365 (1.355-8.356), 0.009** | 6 (10.3)* | **15.096 (3.470-65.678), <0.001** |
| Metronidazole (%) | 11 (37.9) | 12 (20.7)* | 2.328 (0.865-6.267), 0.094 | 7 (12.1)* | **3.869 (1.325-11.292), 0.013** |
| Nitrofurantoin (%) | 5 (17.2) | 3 (5.2) | NA | 6 (10.3) | 1.906 (0.489-7.422), 0.352 |
| Penicillin (%) | 7 (24.1) | 11 (19.0) | 1.314 (0.478-3.616), 0.597 | 9 (15.5) | 1.750 (0.565-5.418), 0.332 |
| Piperacillin/tazobactam (%) | 12 (41.4) | 10 (17.2)* | **5.058 (1.364-18.755), 0.015** | 8 (13.8)* | **5.702 (1.563-20.802), 0.008** |
| Quinolones (%) | 22 (75.9) | 17 (29.3)* | **6.854 (2.289-20.527), 0.001** | 10 (17.2)* | **17.085 (3.963-73.660), <0.001** |
| Trimethoprim/sulfamethoxazole (%) | 7 (24.1) | 4 (6.9) | NA | 3 (5.2) | NA |
| Vancomycin (%) | 21 (72.4) | 8 (13.8)* | **31.663 (4.218-237.689), 0.001** | 4 (6.9) | NA |
| Other antibiotics (%) | 9 (31.0) | 3 (5.2) | NA | 8 (13.8)* | 2.944 (0.952-9.101), 0.061 |
| Selective digestive tract decontamination (%) | 23 (79.3) | 21 (36.2)* | **10.686 (2.429-47.011), 0.002** | 9 (15.5)* | **35.126 (4.700-262.533), 0.001** |
| Treatment related variables; 3 categories | |  |  |  |  |
| Aminoglycosides (%) |  |  |  |  |  |
| 0 days | 19 (65.5) | 50 (86.2) | Reference | 51 (87.9) | Reference |
| 1-3 days | 6 (20.7) | 6 (10.3) | 2.732 (0.648-11.514), 0.171 | 1 (1.7) | NA |
| ≥4 days | 4 (13.8) | 2 (3.4) | NA | 6 (10.3) | NA |
| Amoxicillin/clavulanic acid (%) |  |  |  |  |  |
| 0 days | 24 (82.8) | 46 (79.3) | Reference | 49 (84.5) | Reference |
| 1-3 days | 1 (3.4) | 5 (8.6) | NA | 5 (8.6) | NA |
| ≥4 days | 4 (13.8) | 7 (12.1) | NA | 4 (6.9) | NA |
| Carbapenems (%) |  |  |  |  |  |
| 0 days | 14 (48.3) | 45 (77.6) | Reference | 54 (93.1) | Reference |
| 1-3 days | 2 (6.9) | 1 (1.7) | NA | 2 (3.4) | NA |
| ≥4 days | 13 (44.8) | 12 (20.7) | 3.720 (1.289-10.739), 0.015 | 2 (3.4) | NA |
| Cephalosporins (%) |  |  |  |  |  |
| 0 days | 6 (20.7) | 35 (60.3)* | Reference | 38 (65.5)* | Reference |
| 1-3 days | 8 (27.6) | 6 (10.3)* | **8.662 (1.540-48.735), 0.014** | 10 (17.2)* | **5.234 (1.272-21.533), 0.022** |
| ≥4 days | 15 (51.7) | 17 (29.3)* | **4.367 (1.317-14.478), 0.016** | 10 (17.2)* | **16.211 (3.081-85.300), 0.001** |
| Colistin (%) |  |  |  |  |  |
| 0 days | 25 (86.2) | 51 (87.9) | Reference | 57 (98.3) | Reference |
| 1-3 days | 1 (3.4) | 2 (3.4) | NA | 0 (0) | NA |
| ≥4 days | 3 (10.3) | 5 (8.6) | NA | 1 (1.7) | NA |
| Macrolides (%) |  |  |  |  |  |
| 0 days | 11 (37.9) | 41 (70.7) | Reference | 52 (89.7) | Reference |
| 1-3 days | 1 (3.4) | 4 (6.9) | NA | 2 (3.4) | NA |
| ≥4 days | 17 (58.6) | 13 (22.4) | **4.542 (1.619-12.743), 0.004** | 4 (6.9) | NA |
| Metronidazole (%) |  |  |  |  |  |
| 0 days | 18 (62.1) | 46 (79.3) | Reference | 51 (87.9) | Reference |
| 1-3 days | 3 (10.3) | 4 (6.9) | NA | 2 (3.4) | NA |
| ≥4 days | 8 (27.6) | 8 (13.8) | 2.424 (0.813-7.228), 0.112 | 5 (8.6) | **3.747 (1.173-11.976), 0.026** |
| Penicillin (%) |  |  |  |  |  |
| 0 days | 22 (75.9) | 47 (81.0) | Reference | 49 (84.5) | Reference |
| 1-3 days | 0 (0) | 2 (3.4) | NA | 4 (6.9) | NA |
| ≥4 days | 7 (24.1) | 9 (15.5) | 1.670 (0.571-4.889), 0.349 | 5 (8.6) | 2.813 (0.806-9.819), 0.105 |
| Piperacillin/tazobactam (%) |  |  |  |  |  |
| 0 days | 17 (58.6) | 48 (82.8) | Reference | 50 (86.2) | Reference |
| 1-3 days | 4 (13.8) | 7 (12.1) | NA | 5 (8.6) | NA |
| ≥4 days | 8 (27.6) | 3 (5.2) | NA | 3 (5.2) | NA |
| Quinolones (%) |  |  |  |  |  |
| 0 days | 7 (24.1) | 41 (70.7) | Reference | 48 (82.8) | Reference |
| 1-3 days | 4 (13.8) | 6 (10.3) | NA | 5 (8.6) | NA |
| ≥4 days | 18 (62.1) | 11 (19.0) | **9.314 (2.608-33.265), 0.001** | 5 (8.6) | **42.072 (5.065-349.473), 0.001** |
| Trimethoprim/sulfamethoxazole (%) |  |  |  |  |  |
| 0 days | 22 (75.9) | 54 (93.1) | Reference | 55 (94.8) | Reference |
| 1-3 days | 2 (6.9) | 0 (0) | NA | 1 (1.7) | NA |
| ≥4 days | 5 (17.2) | 4 (6.9) | NA | 2 (3.4) | NA |
| Vancomycin (%) |  |  |  |  |  |
| 0 days | 8 (27.6) | 50 (86.2) | Reference | 54 (93.1) | Reference |
| 1-3 days | 8 (27.6) | 1 (1.7) | NA | 2 (3.4) | NA |
| ≥4 days | 13 (44.8) | 7 (12.1) | **25.137 (2.912-217.002), 0.003** | 2 (3.4) | NA |
| Selective digestive tract decontamination (%) |  |  |  |  |  |
| 0 days | 6 (20.7) | 37 (63.8) | Reference | 49 (84.5) | Reference |
| 1-3 days | 4 (13.8) | 5 (8.6) | NA | 1 (1.7) | NA |
| ≥4 days | 19 (65.5) | 16 (27.6) | **15.564 (2.719-89.080), 0.002** | 8 (13.8) | **42.081 (4.440-398.810), 0.001** |
| Treatment related variables; 4 categories | | |  |  |  |
| Aminoglycosides (%) |  |  |  |  |  |
| 0 days | 19 (65.5) | 50 (86.2) | Reference | 51 (87.9) | Reference |
| 1-3 days | 6 (20.7) | 6 (10.3) | 2.732 (0.648-11.514), 0.171 | 1 (1.7) | NA |
| 4-10 days | 1 (3.4) | 0 (0) | NA | 2 (3.4) | NA |
| ≥11 days | 3 (10.3) | 2 (3.4) | NA | 4 (6.9) | NA |
| Amoxicillin/clavulanic acid (%) |  |  |  |  |  |
| 0 days | 24 (82.8) | 46 (79.3) | Reference | 49 (84.5) | Reference |
| 1-3 days | 1 (3.4) | 5 (8.6) | NA | 5 (8.6) | NA |
| 4-10 days | 4 (13.8) | 6 (10.3) | NA | 3 (5.2) | NA |
| ≥11 days | 0 (0) | 1 (1.7) | NA | 1 (1.7) | NA |
| Carbapenems (%) |  |  |  |  |  |
| 0 days | 14 (48.3) | 45 (77.6) | Reference | 54 (93.1) | Reference |
| 1-3 days | 2 (6.9) | 1 (1.7) | NA | 2 (3.4) | NA |
| 4-10 days | 6 (20.7) | 3 (5.2) | NA | 1 (1.7) | NA |
| ≥11 days | 7 (24.1) | 9 (15.5) | 2.589 (0.778-8.611), 0.121 | 1 (1.7) | NA |
| Cephalosporins (%) |  |  |  |  |  |
| 0 days | 6 (20.7) | 35 (60.3) | Reference | 38 (65.5) | Reference |
| 1-3 days | 8 (27.6) | 6 (10.3) | **8.731 (1.524-50.013), 0.015** | 10 (17.2) | **5.123 (1.240-21.160), 0.024** |
| 4-10 days | 9 (31.0) | 11 (19.0) | **4.449 (1.193-16.584), 0.025** | 9 (15.5) | **9.846 (1.727-54.125), 0.010** |
| ≥11 days | 6 (20.7) | 6 (10.3) | 4.246 (0.991-18.190), 0.051 | 1 (1.7) | NA |
| Colistin (%) |  |  |  |  |  |
| 0 days | 25 (86.2) | 51 (87.9) | Reference | 57 (98.3) | Reference |
| 1-3 days | 1 (3.4) | 2 (3.4) | NA | 0 (0) | NA |
| 4-10 days | 1 (3.4) | 4 (6.9) | NA | 0 (0) | NA |
| ≥11 days | 2 (6.9) | 1 (1.7) | NA | 1 (1.7) | NA |
| Macrolides (%) |  |  |  |  |  |
| 0 days | 11 (37.9) | 41 (70.7) | Reference | 52 (89.7) | Reference |
| 1-3 days | 1 (3.4) | 4 (6.9) | NA | 2 (3.4) | NA |
| 4-10 days | 12 (41.4) | 11 (19.0) | **3.825 (1.254-11.667), 0.018** | 4 (6.9) | NA |
| ≥11 days | 5 (17.2) | 2 (3.4) | NA | 0 (0) | NA |
| Metronidazole (%) |  |  |  |  |  |
| 0 days | 18 (62.1) | 46 (79.3) | Reference | 51 (87.9) | Reference |
| 1-3 days | 3 (10.3) | 4 (6.9) | NA | 2 (3.4) | NA |
| 4-10 days | 4 (13.8) | 5 (8.6) | NA | 4 (6.9) | NA |
| ≥11 days | 4 (13.8) | 3 (5.2) | NA | 1 (1.7) | NA |
| Penicillin (%) |  |  |  |  |  |
| 0 days | 22 (75.9) | 47 (81.0) | Reference | 49 (84.5) | Reference |
| 1-3 days | 0 (0) | 2 (3.4) | NA | 4 (6.9) | NA |
| 4-10 days | 5 (17.2) | 7 (12.1) | 1.540 (0.456-5.198), 0.487 | 5 (8.6) | 1.878 (0.488-7.219), 0.359 |
| ≥11 days | 2 (6.9) | 2 (3.4) | NA | 0 (0) | NA |
| Piperacillin/tazobactam (%) |  |  |  |  |  |
| 0 days | 17 (58.6) | 48 (82.8) | Reference | 50 (86.2) | Reference |
| 1-3 days | 4 (13.8) | 7 (12.1) | NA | 5 (8.6) | NA |
| 4-10 days | 7 (24.1) | 1 (1.7) | NA | 3 (5.2) | NA |
| ≥11 days | 1 (3.4) | 2 (3.4) | NA | 0 (0) | NA |
| Quinolones (%) |  |  |  |  |  |
| 0 days | 7 (24.1) | 41 (70.7) | Reference | 48 (82.8) | Reference |
| 1-3 days | 4 (13.8) | 6 (10.3) | NA | 5 (8.6) | NA |
| 4-10 days | 12 (41.4) | 5 (8.6) | **17.015 (3.194-90.650), 0.001** | 3 (5.2) | NA |
| ≥11 days | 6 (20.7) | 6 (10.3) | **5.239 (1.170-23.461), 0.030** | 2 (3.4) | NA |
| Trimethoprim/sulfamethoxazole (%) |  |  |  |  |  |
| 0 days | 22 (75.9) | 54 (93.1) | Reference | 55 (94.8) | Reference |
| 1-3 days | 2 (6.9) | 0 (0) | NA | 1 (1.7) | NA |
| 4-10 days | 4 (13.8) | 3 (5.2) | NA | 0 (0) | NA |
| ≥11 days | 1 (3.4) | 1 (1.7) | NA | 2 (3.4) | NA |
| Vancomycin (%) |  |  |  |  |  |
| 0 days | 8 (27.6) | 50 (86.2) | Reference | 54 (93.1) | Reference |
| 1-3 days | 8 (27.6) | 1 (1.7) | NA | 2 (3.4) | NA |
| 4-10 days | 3 (10.3) | 3 (5.2) | NA | 1 (1.7) | NA |
| ≥11 days | 10 (34.5) | 4 (6.9) | NA | 1 (1.7) | NA |
| Selective digestive tract decontamination (%) |  |  |  |  |  |
| 0 days | 6 (20.7) | 37 (63.8) | Reference | 49 (84.5) | Reference |
| 1-3 days | 4 (13.8) | 5 (8.6) | NA | 1 (1.7) | NA |
| 4-10 days | 5 (17.2) | 9 (15.5) | 5.549 (0.709-43.444), 0.103 | 5 (8.6) | **14.508 (1.263-166.688), 0.032** |
| ≥11 days | 14 (48.3) | 7 (12.1) | **40.299 (3.834-423.619), 0.002** | 3 (5.2) | NA |

Abbreviations: 95% CI= 95% confidence interval, OR= odds ratio, NA= not applicable, bold= statistically significant, *P*-value <0.05. *= included in multivariable analysis.

**Table 2a.** Patient related clinical variables of case patients with DiversiLab (bioMérieux) clonal cluster B, univariate analysis

| Variables | Cases  (n= 105) | Controls 1&2 (n= 210) | Crude OR  (95% CI), *P*-value | Controls 3&4 (n= 210) | Crude OR  (95% CI), *P*-value |
| --- | --- | --- | --- | --- | --- |
| Basic characteristics |  |  |  |  |  |
| Age, years, median (range) | 60.2 (17-82) | 60.4 (17-90) | 1.001 (0.988-1.014), 0.902 | 61.2 (17-89) | 1.000 (0.986-1.014), 0.989 |
| Male gender (%) | 59 (56.2) | 127 (60.5) | 0.836 (0.517-1.350), 0.463 | 129 (61.4) | 0.790 (0.481-1.299), 0.353 |
| 28-day mortality (%) | 33 (31.4) | 46 (21.9) | 1.716 (0.985-2.990), 0.057 | 28 (13.3) | **3.491 (1.830-6.662), <0.001** |
| 1-year mortality (%) | 66 (62.9) | 66 (31.4) | **3.451 (2.098-5.678), <0.001** | 40 (19.0) | **7.000 (3.887-12.607), <0.001** |
| Transferred from another hospital (%) | 36 (34.3) | 44 (21.0)* | **2.000 (1.171-3.416), 0.011** | 36 (17.1)* | **2.448 (1.427-4.200), 0.001** |
| Length of admission, days, median (range) | 54 (3-341) | 24 (1-258) | **1.021 (1.013-1.029), <0.001** | 12 (1-1102) | **1.018 (1.011-1.025), <0.001** |
| Erasmus MC 1y before VIM-PA (%) | 52 (49.5) | 77 (36.7)* | **1.750 (1.070-2.862), 0.026** | 79 (37.6)* | **1.707 (1.035-2.816), 0.036** |
| Erasmus MC ICU 1y before VIM-PA (%) | 11 (10.5) | 10 (4.8)* | 2.200 (0.934-5.180), 0.071 | 11 (5.2)* | 2.137 (0.888-5.319), 0.089 |
| Surgery (%) | 61 (58.1) | 82 (39.0)* | **2.163 (1.334-3.509), 0.002** | 86 (41.0)* | **2.012 (1.242-3.259), 0.004** |
| Underlying diseases |  |  |  |  |  |
| Cystic fibrosis (%) | 1* (1.0) | 2 (1.0) | NA | 1 (0.5) | NA |
| Chronic respiratory illness (%) | 19 (18.1) | 32 (15.2) | 1.255 (0.651-2.417), 0.498 | 29 (13.8) | 1.455 (0.732-2.889), 0.284 |
| Chronic kidney failure (%) | 4 (3.8) | 2 (1.0) | NA | 8 (3.8) | NA |
| Acute kidney failure; use of CVVH (%) | 23 (21.9) | 10 (4.8)* | **5.978 (2.549-14.022), <0.001** | 6 (2.9)* | **8.916 (3.383-23.497), <0.001** |
| Chronic liver failure (%) | 4 (3.8) | 6 (2.9) | NA | 16 (7.6) | NA |
| Acute liver failure (%) | 0 (0) | 5 (2.4) | NA | 3 (1.4) | NA |
| Chronic problems of the gastrointestinal tract (%) | 14 (13.3) | 21 (10.0) | 1.358 (0.675-2.733), 0.391 | 20 (9.5) | 1.517 (0.704-3.268), 0.288 |
| Acute problems of the gastrointestinal tract (%) | 20 (19.0) | 23 (11.0)* | 1.823 (0.973-3.417), 0.061 | 37 (17.6) | 1.104 (0.597-2.041), 0.752 |
| Auto-immune disease (%) | 5 (4.8) | 14 (6.7) | 0.689 (0.236-2.011), 0.495 | 20 (9.5) | 0.474 (0.173-1.305), 0.149 |
| Human immunodeficiency virus (%) | 0 (0) | 0 (0) | NA | 4 (1.9) | NA |
| Diabetes (%) | 20 (19.0) | 29 (13.8) | 1.478 (0.784-2.784), 0.227 | 40 (19.0) | 1.000 (0.545-1.834), 1.000 |
| Solid organ transplant recipient (%) | 17* (16.3) | 15 (7.1)* | **2.765 (1.243-6.151), 0.013** | 23 (11.0) | 1.661 (0.833-3.310), 0.149 |
| Stem cell/bone marrow transplant recipient (%) | 6* (5.8) | 5 (2.4)* | 4.328 (0.821-22.818), 0.084 | 1 (0.5) | NA |
| Use of immunosuppressive agents (%) | 41 (39.0) | 44 (21.0)* | **2.469 (1.449-4.209), 0.001** | 39 (18.6)* | **2.650 (1.576-4.455), <0.001** |
| Malignancies (%) | 26 (24.8) | 55 (26.2) | 0.920 (0.520-1.625), 0.773 | 69 (32.9) | 0.636 (0.361-1.119), 0.117 |
| Neutropenia, <500 cells/µL (%) | 2 (2.5)** | 4 (2.9)*** | NA | 6 (4.4)**** | NA |
| Endoscopies |  |  |  |  |  |
| Colonoscopy (%) | 6 (5.7) | 8 (3.8) | 1.500 (0.520-4.323), 0.453 | 10 (4.8) | 1.255 (0.403-3.905), 0.695 |
| Sigmoidoscopy (%) | 6 (5.7) | 3 (1.4) | NA | 5 (2.4) | 2.400 (0.732-7.864), 0.148 |
| Endoscopic ultrasound (%) | 6 (5.7) | 23 (11.0) | 0.477 (0.185-1.233), 0.127 | 13 (6.2) | 0.915 (0.330-2.538), 0.864 |
| Gastroscopy (%) | 55 (52.4) | 34 (16.2)* | **7.213 (3.726-13.966), <0.001** | 42 (20.0)* | **4.430 (2.561-7.664), <0.001** |
| ERCP (%) | 12 (11.4) | 11 (5.2)* | **2.530 (1.015-6.307), 0.046** | 9 (4.3)* | **2.841 (1.154-6.992), 0.023** |
| Bronchoscopy (%) | 33 (31.4) | 24 (11.4)* | **3.859 (2.035-7.320), <0.001** | 24 (11.4)* | **3.508 (1.908-6.447), <0.001** |
| Transesophageal Echocardiography (TEE) (%) | 9 (8.6) | 9 (4.3) | 2.000 (0.794-5.038), 0.141 | 8 (3.8)* | 2.250 (0.868-5.832), 0.095 |
| Medical devices |  |  |  |  |  |
| Mechanical ventilation (%) | 92 (87.6) | 151 (71.9)* | **4.164 (1.802-9.623), 0.001** | 137 (65.2)* | **6.061 (2.659-13.819), <0.001** |
| Tracheostomy (%) | 21 (20.0) | 27 (12.9)* | 1.900 (0.942-3.830), 0.073 | 7 (3.3)* | **7.838 (2.941-20.877), <0.001** |
| Extracorporeal membrane oxygenation (%) | 2 (1.9) | 0 (0) | NA | 0 (0) | NA |
| Central venous catheter (%) | 77 (73.3) | 93 (44.3)* | **4.556 (2.443-8.497), <0.001** | 54 (25.7)* | **11.961 (5.693-25.129), <0.001** |

Abbreviations: VIM-PA= Verona Integron-encoded Metallo-β-lactamase (VIM)-positive *Pseudomonas aeruginosa*, 95% CI= 95% confidence interval, OR= odds ratio, y= year, ICU= intensive care unit, CVVH= Continuous Veno-Venous Hemofiltration, ERCP= Endoscopic Retrograde Cholangiopancreatography. *for 1 patient underlying diseases were missing. **for 24 patients information was missing, ***for 74 patients information was missing, ****for 73 patients information was missing.

**Table 2b.** Treatment related variables of case patients with DiversiLab (bioMérieux) clonal cluster B, univariate analysis

| Variables | Cases**  (n= 99) | Controls 1&2 (n= 210) | Crude OR  (95% CI), *P*-value | Controls 3&4 (n= 210) | Crude OR  (95% CI), *P*-value |  |  |
| --- | --- | --- | --- | --- | --- | --- | --- |
| Treatment related variables; yes/no |  |  |  |  |  |  |  |
| Antifungals (%) | 62 (62.6) | 60 (28.6)* | **4.557 (2.562-8.104), <0.001** | 38 (18.1)* | **5.840 (3.382-10.084), <0.001** |  |  |
| Antivirals (%) | 12 (12.1) | 14 (6.7) | 2.000 (0.818-4.893), 0.129 | 13 (6.2)* | 2.072 (0.909-4.724), 0.083 |  |  |
| Aminoglycosides (%) | 37 (37.4) | 41 (19.5) | **2.849 (1.534-5.290), 0.001** | 15 (7.1) | **8.293 (3.839-17.913), <0.001** |  |  |
| Amoxicillin/clavulanic acid (%) | 30 (30.3) | 33 (15.7)* | **2.390 (1.284-4.451), 0.006** | 31 (14.8)* | **2.449 (1.347-4.453), 0.003** |  |  |
| Carbapenems (%) | 38 (38.4) | 41 (19.5) | **2.832 (1.548-5.183), 0.001** | 20 (9.5) | **5.848 (2.961-11.550), <0.001** |  |  |
| Cephalosporins (%) | 74 (74.7) | 95 (45.2) | **3.918 (2.153-7.129), <0.001** | 77 (36.7) | **6.049 (3.189-11.475), <0.001** |  |  |
| Colistin (%) | 12 (12.1) | 11 (5.2)* | **2.593 (1.036-6.492), 0.042** | 11 (5.2)* | **3.347 (1.233-9.088), 0.018** |  |  |
| Macrolides (%) | 39 (39.4) | 41 (19.5)* | **2.722 (1.532-4.838), 0.001** | 17 (8.1)* | **6.333 (3.225-12.437), <0.001** |  |  |
| Metronidazole (%) | 34 (34.3) | 30 (14.3) | **2.765 (1.571-4.867), <0.001** | 23 (11.0)* | **4.766 (2.377-9.555), <0.001** |  |  |
| Nitrofurantoin (%) | 11 (11.1) | 9 (4.3)* | **3.234 (1.179-8.869), 0.023** | 16 (7.6) | 1.480 (0.630-3.476), 0.368 |  |  |
| Penicillin (%) | 20 (20.2) | 35 (16.7) | 1.178 (0.640-2.168), 0.599 | 21 (10.0)* | **2.121 (1.083-4.151), 0.028** |  |  |
| Piperacillin/tazobactam (%) | 33 (33.3) | 36 (17.1)* | **2.413 (1.337-4.355), 0.003** | 22 (10.5)* | **4.150 (2.158-7.981), <0.001** |  |  |
| Quinolones (%) | 65 (65.7) | 65 (31.0) | **5.315 (2.845-9.930), <0.001** | 41 (19.5)* | **7.617 (4.065-14.274), <0.001** |  |  |
| Trimethoprim/sulfamethoxazole (%) | 23 (23.2) | 16 (7.6)* | **3.640 (1.752-7.563), 0.001** | 15 (7.1)* | **3.614 (1.790-7.296), <0.001** |  |  |
| Vancomycin (%) | 50 (50.5) | 37 (17.6) | **5.651 (2.955-10.806), <0.001** | 23 (11.0) | **7.556 (3.904-14.626), <0.001** |  |  |
| Other antibiotics (%) | 21 (21.2) | 22 (10.5)* | **2.337 (1.183-4.618), 0.015** | 20 (9.5)* | **2.688 (1.322-5.466), 0.006** |  |  |
| Selective digestive tract decontamination (%) | 64 (64.6) | 78 (37.1) | **4.192 (2.210-7.950), <0.001** | 45 (21.4) | **8.915 (4.367-18.197), <0.001** |  |  |
| Treatment related variables; 3 categories | |  |  |  |  |  |  |
| Aminoglycosides (%) |  |  |  |  |  |  |  |
| 0 days | 62 (62.6) | 169 (80.5) | Reference | 195 (92.9)* | Reference |  |  |
| 1-3 days | 21 (21.2) | 16 (7.6) | **4.295 (1.913-9.639), <0.001** | 6 (2.9)* | **12.127 (4.014-36.639), <0.001** |  |  |
| ≥4 days | 16 (16.2) | 25 (11.9) | 1.985 (0.931-4.232), 0.076 | 9 (4.3)* | **5.770 (2.162-15.397), <0.001** |  |  |
| Amoxicillin/clavulanic acid (%) |  |  |  |  |  |  |  |
| 0 days | 69 (69.7) | 177 (84.3) | Reference | 179 (85.2) | Reference |  |  |
| 1-3 days | 12 (12.1) | 11 (5.2) | **2.790 (1.134-6.864), 0.026** | 13 (6.2) | **2.387 (1.019-5.593), 0.045** |  |  |
| ≥4 days | 18 (18.2) | 22 (10.5) | **2.168 (1.032-4.557), 0.041** | 18 (8.6) | **2.490 (1.219-5.085), 0.012** |  |  |
| Carbapenems (%) |  |  |  |  |  |  |  |
| 0 days | 61 (61.6) | 169 (80.5) | Reference | 190 (90.5)* | Reference |  |  |
| 1-3 days | 6 (6.1) | 14 (6.7) | 1.200 (0.432-3.403), 0.732 | 7 (3.3)* | 2.121 (0.686-6.558), 0.192 |  |  |
| ≥4 days | 32 (32.3) | 27 (12.9) | **3.920 (1.948-7.889), <0.001** | 13 (6.2)* | **9.427 (3.897-22.803), <0.001** |  |  |
| Cephalosporins (%) |  |  |  |  |  |  |  |
| 0 days | 25 (25.3) | 115 ()54.8 | Reference | 133 (63.3) | Reference |  |  |
| 1-3 days | 22 (22.2) | 31 (14.8) | **3.592 (1.648-7.829), 0.001** | 36 (17.1) | **4.149 (1.899-9.065), <0.001** |  |  |
| ≥4 days | 52 (52.5) | 64 (30.5) | **4.064 (2.152-7.675), <0.001** | 41 (19.5) | **7.242 (3.661-14.325), <0.001** |  |  |
| Colistin (%) |  |  |  |  |  |  |  |
| 0 days | 87 (87.9) | 199 (94.8) | Reference | 199 (94.8) | Reference |  |  |
| 1-3 days | 2 (2.0) | 2 (1.0) | NA | 1 (0.5) | NA |  |  |
| ≥4 days | 10 (10.1) | 9 (4.3) | 2.655 (0.983-7.171), 0.054 | 10 (4.8) | **3.221 (1.074-9.659), 0.037** |  |  |
| Macrolides (%) |  |  |  |  |  |  |  |
| 0 days | 60 (60.6) | 169 (80.5) | Reference | 193 (91.9) | Reference |  |  |
| 1-3 days | 17 (17.2) | 21 (10.0) | **2.324 (1.083-4.986), 0.030** | 7 (3.3) | **6.621 (2.612-16.783), <0.001** |  |  |
| ≥4 days | 22 (22.2) | 20 (9.5) | **3.119 (1.512-6.432), 0.020** | 10 (4.8) | **6.126 (2.695-13.928), <0.001** |  |  |
| Metronidazole (%) |  |  |  |  |  |  |  |
| 0 days | 65 (65.7) | 180 (85.7) | Reference | 187 (89.0) | Reference |  |  |
| 1-3 days | 11 (11.1) | 7 (3.3) | **3.571 (1.367-9.328), 0.009** | 8 (3.8) | **4.254 (1.538-11.765), 0.005** |  |  |
| ≥4 days | 23 (23.2) | 23 (11.0) | **2.478 (1.291-4.755), 0.006** | 15 (7.1) | **5.073 (2.250-11.438), <0.001** |  |  |
| Penicillin (%) |  |  |  |  |  |  |  |
| 0 days | 79 (79.8) | 175 (83.3) | Reference | 189 (90.0) | Reference |  |  |
| 1-3 days | 7 (7.1) | 9 (4.3) | 1.609 (0.575-4.500), 0.365 | 9 (4.3) | 1.716 (0.633-4.657), 0.289 |  |  |
| ≥4 days | 13 (13.1) | 26 (12.4) | 1.016 (0.486-2.122), 0.966 | 12 (5.7) | **2.453 (1.057-5.690), 0.037** |  |  |
| Piperacillin/tazobactam (%) |  |  |  |  |  |  |  |
| 0 days | 66 (66.7) | 174 (82.9) | Reference | 188 (89.5) | Reference |  |  |
| 1-3 days | 16 (16.2) | 15 (7.1) | **2.883 (1.266-6.569), 0.012** | 10 (4.8) | **4.581 (1.827-11.483), 0.001** |  |  |
| ≥4 days | 17 (17.2) | 21 (10.0) | 2.097 (1.001-4.396), 0.050 | 12 (5.7) | **3.821 (1.652-8.836), 0.002** |  |  |
| Quinolones (%) |  |  |  |  |  |  |  |
| 0 days | 34 (34.3) | 145 (69.0)* | Reference | 169 (80.5) | Reference |  |  |
| 1-3 days | 14 (14.1) | 21 (10.0)* | **3.563 (1.495-8.492), 0.004** | 10 (4.8) | **7.134 (2.690-18.920), <0.001** |  |  |
| ≥4 days | 51 (51.5) | 44 (21.0)* | **6.153 (3.141-12.051), <0.001** | 31 (14.8) | **7.751 (4.006-14.998), <0.001** |  |  |
| Trimethoprim/sulfamethoxazole (%) |  |  |  |  |  |  |  |
| 0 days | 76 (76.8) | 194 (92.4) | Reference | 195 (92.9) | Reference |  |  |
| 1-3 days | 5 (5.1) | 4 (1.9) | NA | 1 (0.5) | NA |  |  |
| ≥4 days | 18 (18.2) | 12 (5.7) | **3.676 (1.665-8.118), 0.001** | 14 (6.7) | **3.039 (1.425-6.477), 0.004** |  |  |
| Vancomycin (%) |  |  |  |  |  |  |  |
| 0 days | 49 (49.5) | 173 (82.4)* | Reference | 187 (89.0)* | Reference |  |  |
| 1-3 days | 13 (13.1) | 12 (5.7)* | **3.530 (1.490-8.362), 0.004** | 9 (4.3)* | **4.672 (1.837-11.882), 0.001** |  |  |
| ≥4 days | 37 (37.4) | 25 (11.9)* | **8.023 (3.540-18.182), <0.001** | 14 (6.7)* | **9.932 (4.457-22.132), <0.001** |  |  |
| Selective digestive tract decontamination (%) |  |  |  |  |  |  |  |
| 0 days | 35 (35.4) | 132 (62.9)* | Reference | 165 (78.6)* | Reference |  |  |
| 1-3 days | 10 (10.1) | 14 (6.7)* | **2.835 (1.058-7.596), 0.038** | 16 (7.6)* | **4.943 (1.662-14.701), 0.004** |  |  |
| ≥4 days | 54 (54.5) | 64 (30.5)* | **4.822 (2.383-9.756), <0.001** | 29 (13.8)* | **9.952 (4.768-20.772), <0.001** |  |  |
| Treatment related variables; 4 categories | | |  |  |  |  |  |
| Aminoglycosides (%) |  |  |  |  |  |  |  |
| 0 days | 62 (62.6) | 169 (80.5)* | Reference | 195 (92.9) | Reference |  |  |
| 1-3 days | 21 (21.2) | 16 (7.6)* | **4.564 (1.999-10.423), <0.001** | 6 (2.9) | **11.862 (3.924-35.856), <0.001** |  |  |
| 4-10 days | 7 (7.1) | 18 (8.6)* | 1.044 (0.381-2.864), 0.933 | 6 (2.9) | **4.909 (1.282-18.794), 0.020** |  |  |
| ≥11 days | 9 (9.1) | 7 (3.3)* | **4.491 (1.470-13.725), 0.008** | 3 (1.4) | NA |  |  |
| Amoxicillin/clavulanic acid (%) |  |  |  |  |  |  |  |
| 0 days | 69 (69.7) | 177 (84.3) | Reference | 179 (85.2) | Reference |  |  |
| 1-3 days | 12 (12.1) | 11 (5.2) | **2.746 (1.118-6.747), 0.028** | 13 (6.2) | **2.369 (1.011-5.550), 0.047** |  |  |
| 4-10 days | 17 (17.2) | 22 (10.5) | 2.019 (0.950-4.292), 0.068 | 16 (7.6) | **2.561 (1.234-5.312), 0.012** |  |  |
| ≥11 days | 1 (1.0) | 0 (0.0) | NA | 2 (1.0) | NA |  |  |
| Carbapenems (%) |  |  |  |  |  |  |  |
| 0 days | 61 (61.6) | 169 (80.5)* | Reference | 190 (90.5) | Reference |  |  |
| 1-3 days | 6 (6.1) | 14 (6.7)* | 1.144 (0.399-3.278), 0.802 | 7 (3.3) | 2.121 (0.685-6.565), 0.192 |  |  |
| 4-10 days | 15 (15.2) | 21 (10.0)* | **2.475 (1.104-5.549), 0.028** | 10 (4.8) | **6.203 (2.170-17.737), 0.001** |  |  |
| ≥11 days | 17 (17.2) | 6 (2.9)* | **8.749 (3.026-25.294), <0.001** | 3 (1.4) | NA |  |  |
| Cephalosporins (%) |  |  |  |  |  |  |  |
| 0 days | 25 (25.3) | 115 (54.8) | Reference | 13 (6.2) | Reference |  |  |
| 1-3 days | 22 (22.2) | 31 (14.8) | **3.661 (1.661-8.071), 0.001** | 36 (17.1) | **4.609 (2.060-10.310), <0.001** |  |  |
| 4-10 days | 35 (35.4) | 50 (23.8) | **3.478 (1.772-6.827), <0.001** | 34 (16.2) | **5.579 (2.707-11.498), <0.001** |  |  |
| ≥11 days | 17 (17.2) | 14 (6.7) | **6.628 (2.545-17.263), <0.001** | 7 (3.3) | **16.900 (5.206-54.857), <0.001** |  |  |
| Colistin (%) |  |  |  |  |  |  |  |
| 0 days | 87 (87.9) | 199 (94.8) | Reference | 199 (94.8) | Reference |  |  |
| 1-3 days | 2 (2.0) | 2 (1.0) | NA | 1 (0.5) | NA |  |  |
| 4-10 days | 4 (4.0) | 4 (1.9) | NA | 4 (1.9) | NA |  |  |
| ≥11 days | 6 (6.1) | 5 (2.4) | 3.333 (0.797-13.948), 0.099 | 6 (2.9) | 2.947 (0.800-10.857), 0.104 |  |  |
| Macrolides (%) |  |  |  |  |  |  |  |
| 0 days | 60 (60.6) | 169 (80.5) | Reference | 193 (91.9) | Reference |  |  |
| 1-3 days | 17 (17.2) | 21 (10.0) | **2.308 (1.075-4.956), 0.032** | 7 (3.3) | **6.923 (2.651-18.082), <0.001** |  |  |
| 4-10 days | 16 (16.2) | 16 (7.6) | **2.947 (1.297-6.692), 0.010** | 10 (4.8) | **4.337 (1.833-10.264), 0.001** |  |  |
| ≥11 days | 6 (6.1) | 4 (1.9) | NA | 0 (0.0) | NA |  |  |
| Metronidazole (%) |  |  |  |  |  |  |  |
| 0 days | 65 (6.7) | 180 (85.7)* | Reference | 187 (89.0) | Reference |  |  |
| 1-3 days | 11 (11.1) | 7 (3.3)* | **3.593 (1.360-9.493), 0.010** | 8 (3.8) | **5.523 (1.733-17.605), 0.004** |  |  |
| 4-10 days | 11 (11.1) | 18 (8.6)* | 1.468 (0.670-3.218), 0.338 | 14 (6.7) | 2.153 (0.850-5.454), 0.106 |  |  |
| ≥11 days | 12 (12.1) | 5 (2.4)* | **11.148 (2.420-51.358), 0.002** | 1 (0.5) | NA |  |  |
| Penicillin (%) |  |  |  |  |  |  |  |
| 0 days | 79 (79.8) | 180 (85.7) | Reference | 189 (90.0) | Reference |  |  |
| 1-3 days | 7 (7.1) | 7 (3.3) | 1.578 (0.564-4.414), 0.385 | 9 (4.3) | 1.785 (0.652-4.890), 0.260 |  |  |
| 4-10 days | 6 (6.1) | 18 (8.6) | 0.747 (0.288-1.936), 0.548 | 9 (4.3) | 1.495 (0.504-4.434), 0.469 |  |  |
| ≥11 days | 7 (7.1) | 5 (2.4) | 1.601 (0.543-4.723), 0.394 | 3 (1.4) | NA |  |  |
| Piperacillin/tazobactam (%) |  |  |  |  |  |  |  |
| 0 days | 66 (66.7) | 174 (82.9) | Reference | 188 (89.5) | Reference |  |  |
| 1-3 days | 16 (16.2) | 15 (7.1) | **2.844 (1.265-6.571), 0.012** | 10 (4.8) | **4.605 (1.826-11.613), 0.001** |  |  |
| 4-10 days | 13 (13.1) | 16 (7.6) | 2.097 (0.918-4.788), 0.079 | 9 (4.3) | **3.729 (1.448-9.607), 0.006** |  |  |
| ≥11 days | 4 (4.0) | 5 (2.4) | NA | 3 (1.4) | NA |  |  |
| Quinolones (%) |  |  |  |  |  |  |  |
| 0 days | 34 (34.3) | 145 (69.0) | Reference | 169 (80.5) | Reference |  |  |
| 1-3 days | 14 (14.1) | 21 (10.0) | **3.908 (1.612-9.475), 0.003** | 10 (4.8) | **7.154 (2.694-18.996), <0.001** |  |  |
| 4-10 days | 26 (26.3) | 30 (14.3) | **4.295 (2.011-9.171), <0.001** | 17 (8.1) | **7.184 (3.240-15.929), <0.001** |  |  |
| ≥11 days | 25 (25.3) | 14 (6.7) | **10.694 (4.222-27.083), <0.001** | 14 (6.7) | **8.451 (3.637-19.640), <0.001** |  |  |
| Trimethoprim/sulfamethoxazole (%) |  |  |  |  |  |  |  |
| 0 days | 76 (76.8) | 194 (92.4) | Reference | 195 (92.9) | Reference |  |  |
| 1-3 days | 5 (5.1) | 4 (1.9) | NA | 1 (0.5) | NA |  |  |
| 4-10 days | 5 (5.1) | 5 (2.4) | 2.456 (0.684-8.818), 0.168 | 4 (1.9) | NA |  |  |
| ≥11 days | 13 (13.1) | 7 (3.3) | **4.599 (1.705-12.406), 0.003** | 10 (4.8) | **3.187 (1.309-7.759), 0.011** |  |  |
| Vancomycin (%) |  |  |  |  |  |  |  |
| 0 days | 49 (49.5) | 173 (82.4) | Reference | 187 (89.0) | Reference |  |  |
| 1-3 days | 13 (13.1) | 12 (5.7) | **3.390 (1.430-8.034), 0.006** | 9 (4.3) | **4.514 (1.783-11.427), 0.001** |  |  |
| 4-10 days | 19 (19.2) | 21 (10.0) | **4.474 (1.945-11.587), 0.001** | 10 (4.8) | **7.928 (3.094-20.318), <0.001** |  |  |
| ≥11 days | 18 (18.2) | 4 (1.9) | NA | 4 (1.9) | NA |  |  |
| Selective digestive tract decontamination (%) |  |  |  |  |  |  |  |
| 0 days | 35 (35.4) | 132 (62.9) | Reference | 165 (78.6) | Reference |  |  |
| 1-3 days | 10 (10.1) | 14 (6.7) | 2.433 (0.913-6.538), 0.075 | 16 (7.6) | **4.386 (1.475-13.041), 0.008** |  |  |
| 4-10 days | 15 (15.2) | 34 (16.2) | 1.922 (0.768-4.808), 0.163 | 17 (8.1) | **5.142 (2.006-13.181), 0.001** |  |  |
| ≥11 days | 39 (39.4) | 30 (14.3) | **10.530 (4.105-27.008), <0.001** | 12 (5.7) | **14.980 (6.259-35-851), <0.001** |  |  |

Abbreviations: 95% CI= 95% confidence interval, OR= odds ratio, NA= not applicable, bold= statistically significant, *P*-value <0.05. *= included in multivariable analysis, ** for six cases information about antibiotic use was missing.
