# Supplementary material for: VIM-positive Pseudomonas aeruginosa in a large tertiary care hospital: matched case-control studies and a network analysis
Source: Antimicrob Resist Infect Control. 2018 Feb 27;7:32. doi: 10.1186/s13756-018-0325-1 (PMC5828133; doi:10.1186/s13756-018-0325-1)
Supplement: Supplementary file 5 — Text file: Subgroup analysis ICU or non-ICU as ward of acquisition of VIM-PA, univariate analysis. Variables with an asterisk (*) were selected for multivariable analysis. (DOCX 83 kb) [file 13756_2018_325_MOESM5_ESM.docx]

**Additional file 5: Text file:** Subgroup analysis ICU or non-ICU as ward of acquisition of VIM-positive *Pseudomonas aeruginosa*, univariate analysis.

**Table 1a.** Patient related clinical variables of case patients with the intensive care as ward of acquisition, univariate analysis

| Variables | Cases  (n= 90) | Controls 1&2 (n= 180) | Crude OR  (95% CI), *P*-value | Controls 3&4 (n= 180) | Crude OR  (95% CI), *P*-value |
| --- | --- | --- | --- | --- | --- |
| Basic characteristics |  |  |  |  |  |
| Age, years, median (range) | 58.9 (17-81) | 60.6 (17-86) | 0.994 (0.980-1.009), 0.454 | 62.4 (17-89) | 0.991 (0.975-1.006), 0.243 |
| Male gender (%) | 52 (57.8) | 118 (65.6) | 0.718 (0.426-1.211), 0.214 | 106 (58.9) | 0.952 (0.559-1.621), 0.856 |
| 28-day mortality (%) | 35 (38.9) | 54 (30.0) | 1.517 (0.844-2.623), 0.136 | 31 (17.2) | **3.356 (1.793-6.280), <0.001** |
| 1-year mortality (%) | 59 (65.6) | 65 (36.1) | **3.413 (1.952-5.968), <0.001** | 49 (27.2) | **5.319 (2.909-9.726), <0.001** |
| Transferred from another hospital (%) | 39 (43.3) | 42 (23.3)* | **2.511 (1.442-4.374), 0.001** | 35 (19.4)* | **2.818 (1.646-4.822), <0.001** |
| Median length of admission (range) | 54 (3-341) | 26 (1-216) | **1.024 (1.014-1.034), <0.001** | 16 (1-1102) | **1.013 (1.007-1.020), <0.001** |
| Erasmus MC 1y before VIM-PA (%) | 31 (34.4) | 60 (33.3) | 1.048 (0.624-1.758), 0.860 | 67 (37.2) | 0.882 (0.514-1.513), 0.648 |
| Erasmus MC ICU 1y before VIM-PA (%) | 6 (6.7) | 10 (5.6) | 1.200 (0.436-3.302), 0.724 | 10 (5.6) | 1.233 (0.515-3.663), 0.706 |
| Surgery (%) | 49 (54.4) | 93 (51.7) | 1.124 (0.669-1.890), 0.659 | 82 (45.6) | 1.403 (0.854-2.305), 0.181 |
| Underlying diseases |  |  |  |  |  |
| Cystic fibrosis (%) | 0* (0) | 1 (0.6) | NA | 0 (0) | NA |
| Chronic respiratory illness (%) | 18 (20.0) | 28 (15.6) | 1.384 (0.703-2.724), 0.347 | 30 (16.7) | 1.2668 (0.650-2.472), 0.486 |
| Chronic kidney failure (%) | 3 (3.3) | 2 (1.1) | NA | 11 (6.1) | NA |
| Acute kidney failure; use of CVVH (%) | 25 (27.8) | 15 (8.3)* | **3.835 (1.915-7.680), <0.001** | 7 (3.9)* | **9.430 (3.598-24.716), <0.001** |
| Chronic liver failure (%) | 4 (4.4) | 2 (1.1) | NA | 10 (5.6) | NA |
| Acute liver failure (%) | 0 (0) | 4 (2.2) | NA | 2 (1.1) | NA |
| Chronic problems of the gastrointestinal tract (%) | 10 (11.1) | 19 (10.6) | 1.055 (0.484-2.299), 0.894 | 14 (7.8) | 1.547 (0.625-3.829), 0.346 |
| Acute problems of the gastrointestinal tract (%) | 19 (21.1) | 25 (13.9) | 1.592 (0.846-2.996), 0.149 | 33 (18.3) | 1.203 (0.629-2.301), 0.577 |
| Auto-immune disease (%) | 5 (5.6) | 11 (6.1) | 0.897 (0.291-2.769), 0.850 | 18 (10.0) | 0.529 (0.189-1.476), 0.224 |
| Human immunodeficiency virus (%) | 0 (0) | 2 (1.1) | NA | 2 (1.1) | NA |
| Diabetes (%) | 13 (14.4) | 20 (11.1) | 1.367 (0.635-2.944), 0.424 | 33 (18.3) | 0.752 (0.374-1.511), 0.423 |
| Solid organ transplant recipient (%) | 13** (14.6) | 12 (6.7)* | 2.167 (0.989-4.748), 0.053 | 22 (12.2) | 1.300 (0.601-2.812), 0.505 |
| Stem cell/bone marrow transplant recipient (%) | 5** (5.6) | 3 (1.7) | NA | 0 (0) | NA |
| Use of immunosuppressive agents (%) | 37 (41.1) | 61 (33.9) | 1.352 (0.807-2.264), 0.252 | 39 (21.7)* | **2.396 (1.395-4.116), 0.002** |
| Malignancies (%) | 17 (18.9) | 36 (20.0) | 0.934 (0.497-1.755), 0.831 | 60 (33.3)* | **0.426 (0.222-0.816), 0.010** |
| Neutropenia, <500 cells/µL (%) | 2*** (2.9) | 2**** (1.6) | NA | 5***** (4.3) | NA |
| Endoscopies |  |  |  |  |  |
| Colonoscopy (%) | 5 (5.6) | 4 (2.2) | NA | 6 (3.3) | 1.766 (0.500-6.237), 0.377 |
| Sigmoidoscopy (%) | 5 (5.6) | 4 (2.2) | NA | 5 (2.8) | 2.000 (0.579-6.908), 0.273 |
| Endoscopic ultrasound (%) | 2 (2.2) | 13 (7.2) | NA | 13 (7.2) | NA |
| Gastroscopy (%) | 48 (53.3) | 25 (13.9)* | **7.571 (3.791-15.120), <0.001** | 38 (21.1)* | **4.038 (2.284-7.138), <0.001** |
| ERCP (%) | 11 (12.2) | 6 (3.3)* | **4.132 (1.426-11.976), 0.009** | 6 (3.3)* | **4.132 (1.426-11.976), 0.009** |
| Bronchoscopy (%) | 31 (34.4) | 25 (13.9*) | **3.444 (1.800-6.587), <0.001** | 19 (10.6)* | **4.438 (2.255-8.735), <0.001** |
| Transesophageal Echocardiography (TEE) (%) | 11 (12.2) | 7 (3.9)* | **3.143 (1.218-8.107), 0.018** | 8 (4.4)* | **2.750 (1.106-6.837), 0.029** |
| Medical devices |  |  |  |  |  |
| Mechanical ventilation (%) | 87 (96.7) | 155 (86.1)* | **4.720 (1.377-16.175), 0.014** | 130 (72.2)* | **9.430 (3.598-24.716), <0.001** |
| Tracheostomy (%) | 19 (21.1) | 29 (16.1) | 1.486 (0.735-3.007), 0.271 | 5 (2.8)* | **9.144 (3.103-26.948), <0.001** |
| Extracorporeal membrane oxygenation (%) | 2 (2.2) | 0 (0) | NA | 0 (0) | NA |
| Central venous catheter (%) | 73 (81.1) | 96 (53.3)* | **4.589 (2.272-9.270), <0.001** | 57 (31.7)* | **11.832 (5.356-26.139), <0.001** |

Abbreviations: VIM-PA= Verona Integron-encoded Metallo-β-lactamase (VIM)-positive *Pseudomonas aeruginosa*, 95% CI= 95% confidence interval, OR= odds ratio, y= year, ICU= intensive care unit, CVVH= Continuous Veno-Venous Hemofiltration, ERCP= Endoscopic Retrograde Cholangiopancreatography, NA= not applicable. *Included in multivariable analysis, **for 1 patient information was missing, ***for 20 patients information was missing, ****for 57 patients information was missing, ***** for 64 patients information was missing.**Table 1b.** Treatment related variables of case patients with the intensive care as ward of acquisition, univariate analysis

| Variables | Cases  (n= 86**) | Controls 1&2 (n= 180) | Crude OR  (95% CI), *P*-value | Controls 3&4 (n= 180) | Crude OR  (95% CI), *P*-value |  |  |
| --- | --- | --- | --- | --- | --- | --- | --- |
| Treatment related variables; yes/no |  |  |  |  |  |  |  |
| Antifungals (%) | 58 (67.4) | 60 (33.3)* | **4.879 (2.542-9.361), <0.001** | 31 (17.2)* | **6.691 (3.701-12.095), <0.001** |  |  |
| Antivirals (%) | 12 (14.0) | 16 (8.9) | 1.658 (0.711-3.869), 0.242 | 9 (5.0)* | **2.667 (1.124-6.329), 0.026** |  |  |
| Aminoglycosides (%) | 34 (39.5) | 43 (23.9) | **2.298 (1.235-4.275), 0.009** | 15 (8.3)* | **6.779 (3.233-14.217), <0.001** |  |  |
| Amoxicillin/clavulanic acid (%) | 24 (27.9) | 32 (17.8) | 1.863 (0.951-3.650), 0.070 | 26 (14.4)* | **2.228 (1.169-4.248), 0.015** |  |  |
| Carbapenems (%) | 38 (44.2) | 39 (21.7) | **3.008 (1.630-5.552), <0.001** | 18 (10.0) | **6.028 (3.059-11.876), <0.001** |  |  |
| Cephalosporins (%) | 71 (82.6) | 109 (60.6)* | **2.798 (1.463-5.348), 0.002** | 76 (42.2) | **7.816 (3.647-16.749), <0.001** |  |  |
| Colistin (%) | 10 (11.6) | 14 (7.8) | 1.480 (0.630-3.476), 0.368 | 7 (3.9)* | **3.735 (1.266-11.017), 0.017** |  |  |
| Macrolides (%) | 42 (48.8) | 56 (31.1) | **2.048 (1.178-3.563), 0.011** | 21 (11.7) | **6.372 (3.252-12.484), <0.001** |  |  |
| Metronidazole (%) | 32 (37.2) | 35 (19.4)* | **2.166 (1.244-3.770), 0.006** | 26 (14.4) | **3.491 (1.830-6.662), <0.001** |  |  |
| Nitrofurantoin (%) | 11 (12.8) | 8 (4.4)* | **3.869 (1.325-11.292), 0.013** | 17 (9.4) | 1.391 (0.588-3.291), 0.453 |  |  |
| Penicillin (%) | 20 (23.3) | 31 (17.2) | 1.358 (0.731-2.523), 0.333 | 23 (12.8) | 1.932 (0.995-3.752), 0.052 |  |  |
| Piperacillin/tazobactam (%) | 31 (36.0) | 30 (16.7)* | **3.166 (1.611-6.223), 0.001** | 28 (15.6)* | **3.135 (1.633-6.018), 0.001** |  |  |
| Quinolones (%) | 60 (69.8) | 68 (37.8) | **4.626 (2.394-8.938), <0.001** | 32 (17.8) | **7.720 (4.113-14.490), <0.001** |  |  |
| Trimethoprim/sulfamethoxazole (%) | 23 (26.7) | 16 (8.9)* | **4.145 (1.885-9.114), <0.001** | 11 (6.1)* | **4.465 (2.120-9.402), <0.001** |  |  |
| Vancomycin (%) | 47 (54.7) | 36 (20.0) | **5.621 (2.858-11.056), <0.001** | 17 (9.4) | **8.409 (4.229-16.720), <0.001** |  |  |
| Other antibiotics (%) | 20 (23.3) | 17 (9.4)* | **2.953 (1.425-6.121), 0.004** | 23 (12.8)* | **2.000 (1.033-3.872), 0.040** |  |  |
| Selective digestive tract decontamination (%) | 66 (76.7) | 94 (52.2) | **3.935 (1.861-8.321), <0.001** | 42 (23.3) | **13.763 (5.891-32.153), <0.001** |  |  |
| Treatment related variables; 3 categories | |  |  |  |  |  |  |
| Aminoglycosides (%) |  |  |  |  |  |  |  |
| 0 days | 52 (60.5) | 137 (76.1) | Reference | 165 (91.7) | Reference |  |  |
| 1-3 days | 22 (25.6) | 19 (10.6) | **3.474 (1.600-7.544), 0.002** | 4 (2.2) | NA |  |  |
| ≥4 days | 12 (14.0) | 24 (13.3) | 1.425 (0.633-3.210), 0.392 | 11 (6.1) | **3.011 (1.126-8.051), 0.028** |  |  |
| Amoxicillin/clavulanic acid (%) |  |  |  |  |  |  |  |
| 0 days | 62 (72.1) | 148 (82.2) | Reference | 154 (85.6) | Reference |  |  |
| 1-3 days | 11 (12.8) | 13 (7.2) | 2.190 (0.853-5.624), 0.103 | 11 (6.1) | 2.331 (0.973-5.582), 0.058 |  |  |
| ≥4 days | 13 (15.1) | 19 (10.6) | 1.678 (0.754-3.732), 0.205 | 15 (8.3) | 2.142 (0.940-4.880), 0.070 |  |  |
| Carbapenems (%) |  |  |  |  |  |  |  |
| 0 days | 48 (55.8) | 141 (78.3) | Reference | 162 (90.0)* | Reference |  |  |
| 1-3 days | 6 (7.0) | 10 (5.6) | 1.591 (0.538-4.701), 0.401 | 5 (2.8)* | 2.684 (0.744-9.680), 0.131 |  |  |
| ≥4 days | 32 (37.2) | 29 (16.1) | **3.765 (1.870-7.581), <0.001** | 13 (7.2)* | **7.933 (3.476-18.107), <0.001** |  |  |
| Cephalosporins (%) |  |  |  |  |  |  |  |
| 0 days | 15 (17.4) | 71 (39.4) | Reference | 104 (57.8)* | Reference |  |  |
| 1-3 days | 20 (23.3) | 32 (17.8) | **2.781 (1.194-6.479), 0.018** | 37 (20.6)* | **4.883 (2.015-11.833), <0.001** |  |  |
| ≥4 days | 51 (59.3) | 77 (42.8) | **2.803 (1.434-5.479), 0.003** | 39 (21.7)* | **9.920 (4.424-22.242), <0.001** |  |  |
| Colistin (%) |  |  |  |  |  |  |  |
| 0 days | 76 (88.4) | 166 (92.2) | Reference | 173 (96.1) | Reference |  |  |
| 1-3 days | 1 (1.2) | 4 (2.2) | NA | 1 (0.6) | NA |  |  |
| ≥4 days | 9 (10.5) | 10 (5.6) | 1.841 (0.723-4.686), 0.201 | 6 (3.3) | **4.165 (1.271-13.653), 0.018** |  |  |
| Macrolides (%) |  |  |  |  |  |  |  |
| 0 days | 44 (51.2) | 124 (68.9)* | Reference | 159 (88.3)* | Reference |  |  |
| 1-3 days | 13 (15.1) | 29 (16.1)* | 1.136 (0.525-2.461), 0.746 | 9 (5.0)* | **4.439 (1.765-11.165), 0.002** |  |  |
| ≥4 days | 29 (33.7) | 27 (15.0)* | **3.287 (1.611-6.707), 0.001** | 12 (6.7)* | **8.086 (3.548-18.426), <0.001** |  |  |
| Metronidazole (%) |  |  |  |  |  |  |  |
| 0 days | 54 (62.8) | 145 (80.6) | Reference | 154 (85.6)* | Reference |  |  |
| 1-3 days | 9 (10.5) | 12 (6.7) | 1.863 (0.735-4.721), 0.190 | 12 (6.7)* | 2.188 (0.829-5.773), 0.114 |  |  |
| ≥4 days | 23 (26.7) | 23 (12.8) | **2.298 (1.228-4.299), 0.009** | 14 (7.8)* | **4.469 (2.076-9.619), <0.001** |  |  |
| Penicillin (%) |  |  |  |  |  |  |  |
| 0 days | 66 (76.7) | 149 (82.8) | Reference | 157 (87.2) | Reference |  |  |
| 1-3 days | 8 (9.3) | 10 (5.6) | 1.678 (0.639-4.411), 0.294 | 10 (5.6) | 1.720 (0.674-4.392), 0.257 |  |  |
| ≥4 days | 12 (14.0) | 21 (11.7) | 1.200 (0.559-2.574), 0.640 | 13 (7.2) | 2.130 (0.898-5.054), 0.086 |  |  |
| Piperacillin/tazobactam (%) |  |  |  |  |  |  |  |
| 0 days | 55 (64.0) | 150 (83.3) | Reference | 152 (84.4) | Reference |  |  |
| 1-3 days | 13 (15.1) | 15 (8.3) | **2.760 (1.129-6.746), 0.026** | 13 (7.2) | **2.842 (1.152-7.010), 0.023** |  |  |
| ≥4 days | 18 (20.9) | 15 (8.3) | **3.507 (1.561-7.879), 0.002** | 15 (8.3) | **3.378 (1.501-7.602), 0.003** |  |  |
| Quinolones (%) |  |  |  |  |  |  |  |
| 0 days | 26 (30.2) | 112 (62.2) | Reference | 148 (82.2) | Reference |  |  |
| 1-3 days | 12 (14.0) | 26 (14.4) | 2.380 (0.961-5.891), 0.061 | 11 (6.1) | **4.341 (1.672-11.271), 0.003** |  |  |
| ≥4 days | 48 (55.8) | 42 (23.3) | **5.919 (2.902-12.073), <0.001** | 21 (11.7) | **9.695 (4.721-19.910), <0.001** |  |  |
| Trimethoprim/sulfamethoxazole (%) |  |  |  |  |  |  |  |
| 0 days | 63 (73.3) | 164 (91.1) | Reference | 169 (93.9) | Reference |  |  |
| 1-3 days | 4 (4.7) | 4 (2.2) | NA | 2 (1.1) | NA |  |  |
| ≥4 days | 19 (22.1) | 12 (6.7) | **4.719 (1.943-11.460), 0.001** | 9 (5.0) | **4.581 (1.999-10.496), <0.001** |  |  |
| Vancomycin (%) |  |  |  |  |  |  |  |
| 0 days | 39 (45.3) | 144 (80.0)* | Reference | 163 (90.6)* | Reference |  |  |
| 1-3 days | 18 (20.9) | 13 (7.2)* | **4.683 (2.089-10.497), <0.001** | 7 (3.9)* | **6.912 (2.642-18.080), <0.001** |  |  |
| ≥4 days | 29 (33.7) | 23 (12.8)* | **6.870 (2.930-16.107), <0.001** | 10 (5.6)* | **9.806 (3.976-24.182), <0.001** |  |  |
| Selective digestive tract decontamination (%) |  |  |  |  |  |  |  |
| 0 days | 20 (23.3) | 86 (47.8) | Reference | 138 (76.7)* | Reference |  |  |
| 1-3 days | 10 (11.6) | 15 (8.3) | **3.437 (1.144-10.322), 0.028** | 13 (7.2)* | **8.330 (2.575-26.946), <0.001** |  |  |
| ≥4 days | 56 (65.1) | 79 (43.9) | **4.071 (1.870-8.860), <0.001** | 29 (16.1)* | **15.206 (6.374-36.277), <0.001** |  |  |
| Treatment related variables; 4 categories | | |  |  |  |  |  |
| Aminoglycosides (%) |  |  |  |  |  |  |  |
| 0 days | 52 (60.5) | 137 (76.1)* | Reference | 165 (91.7) | Reference |  |  |
| 1-3 days | 22 (25.6) | 19 (10.6)* | **3.568 (1.635-7.786), 0.001** | 4 (2.2) | NA |  |  |
| 4-10 days | 6 (7.0) | 16 (8.9)* | 0.936 (0.316-2.778), 0.905 | 6 (3.3) | 3.378 (0.838-13.620), 0.087 |  |  |
| ≥11 days | 6 (7.0) | 8 (4.4)* | 2.381 (0.749-7.565), 0.141 | 5 (2.8) | 2.769 (0.821-9.336), 0.101 |  |  |
| Amoxicillin/clavulanic acid (%) |  |  |  |  |  |  |  |
| 0 days | 62 (72.1) | 148 (82.2) | Reference | 154 (85.6) | Reference |  |  |
| 1-3 days | 11 (12.8) | 13 (7.2) | 2.239 (0.868-5.771), 0.095 | 11 (6.1) | 2.370 (0.988-5.685), 0.053 |  |  |
| 4-10 days | 13 (15.1) | 18 (10.0) | 1.816 (0.803-4.107), 0.152 | 13 (7.2) | **2.357 (1.014-5.479), 0.046** |  |  |
| ≥11 days | 0 (0) | 1 (1.1) | NA | 2 (1.1) | NA |  |  |
| Carbapenems (%) |  |  |  |  |  |  |  |
| 0 days | 48 (55.8) | 141 (78.3)* | Reference | 162 (90.0) | Reference |  |  |
| 1-3 days | 6 (7.0) | 10 (5.6)* | 1.587 (0.537-4.692), 0.404 | 5 (2.8) | 2.684 (0.744-9.680), 0.131 |  |  |
| 4-10 days | 17 (19.8) | 20 (11.1)* | **3.003 (1.337-6.743), 0.008** | 10 (5.6) | **5.775 (2.204-15.133), <0.001** |  |  |
| ≥11 days | 15 (17.4) | 9 (5.0)* | **5.227 (2.049-13.332), 0.001** | 3 (1.7) | NA |  |  |
| Cephalosporins (%) |  |  |  |  |  |  |  |
| 0 days | 15 (17.4) | 71 (39.4) | Reference | 104 (57.8) | Reference |  |  |
| 1-3 days | 20 (23.3) | 32 (17.8) | **2.722 (1.150-6.440), 0.023** | 37 (20.6) | **6.203 (2.444-15.744), <0.001** |  |  |
| 4-10 days | 32 (37.2) | 63 (35.0) | **2.136 (1.040-4.386), 0.039** | 34 (18.9) | **6.505 (2.820-15.005), <0.001** |  |  |
| ≥11 days | 19 (22.1) | 14 (7.8) | **5.544 (2.211-13.903), <0.001** | 5 (2.8) | **44.966 (10.486-192.824), <0.001** |  |  |
| Colistin (%) |  |  |  |  |  |  |  |
| 0 days | 76 (88.4) | 166 (92.2) | Reference | 173 (96.1) | Reference |  |  |
| 1-3 days | 1 (1.2) | 4 (2.2) | NA | 1 (0.6) | NA |  |  |
| 4-10 days | 2 (2.3) | 7 (3.9) | NA | 1 (0.6) | NA |  |  |
| ≥11 days | 7 (8.1) | 3 (1.7) | NA | 5 (2.8) | **3.500 (1.025-11.956), 0.046** |  |  |
| Macrolides (%) |  |  |  |  |  |  |  |
| 0 days | 44 (51.2) | 124 (68.9) | Reference | 159 (88.3) | Reference |  |  |
| 1-3 days | 13 (15.1) | 29 (16.1) | 1.130 (0.521-2.451), 0.757 | 9 (5.0) | 4.420 (1.755-11.131), 0.002 |  |  |
| 4-10 days | 22 (25.6) | 22 (12.2) | **3.169 (1.431-7.018), 0.004** | 11 (6.1) | **6.748 (2.821-16.143), <0.001** |  |  |
| ≥11 days | 7 (8.1) | 5 (2.8) | **3.623 (1.107-11.857), 0.033** | 1 (0.6) | **NA** |  |  |
| Metronidazole (%) |  |  |  |  |  |  |  |
| 0 days | 54 (62.8) | 145 (80.6) | Reference | 154 (85.6) | Reference |  |  |
| 1-3 days | 9 (10.5) | 12 (6.7) | 1.894 (0.745-4.817), 0.180 | 12 (6.7) | 2.674 (0.929-7.696), 0.068 |  |  |
| 4-10 days | 9 (10.5) | 18 (10.0) | 1.058 (0.547-2.447), 0.896 | 14 (7.8) | 1.559 (0.612-3.973), 0.352 |  |  |
| ≥11 days | 14 (16.3) | 5 (2.8) | **9.032 (2.546-32.032), 0.001** | 0 (0) | **NA** |  |  |
| Penicillin (%) |  |  |  |  |  |  |  |
| 0 days | 66 (76.7) | 149 (82.8) | Reference | 157 (87.2) | Reference |  |  |
| 1-3 days | 8 (9.3) | 10 (5.6) | 1.659 (0.631-4.364), 0.305 | 10 (5.6) | 1.643 (0.645-4.184), 0.298 |  |  |
| 4-10 days | 7 (8.1) | 14 (7.8) | 1.064 (0.417-2.720), 0.896 | 12 (6.7) | 1.345 (0.489-3.698), 0.545 |  |  |
| ≥11 days | 5 (5.8) | 7 (3.9) | 1.489 (0.443-4.999), 0.520 | 1 (0.6) | NA |  |  |
| Piperacillin/tazobactam (%) |  |  |  |  |  |  |  |
| 0 days | 55 (64.0) | 150 (83.3) | Reference | 152 (84.4) | Reference |  |  |
| 1-3 days | 13 (15.1) | 15 (8.3) | **2.642 (1.076-6.484), 0.034** | 13 (7.2) | **2.792 (1.133-6.880), 0.026** |  |  |
| 4-10 days | 15 (17.4) | 11 (6.1) | **4.099 (1.600-10.503), 0.003** | 11 (6.1) | **4.002 (1.574-10.173), 0.004** |  |  |
| ≥11 days | 3 (3.5) | 4 (2.2) | NA | 4 (2.2) | NA |  |  |
| Quinolones (%) |  |  |  |  |  |  |  |
| 0 days | 26 (30.2) | 112 (62.2)* | Reference | 148 (82.2)* | Reference |  |  |
| 1-3 days | 12 (14.0) | 26 (14.4)* | 2.356 (0.948-5.853), 0.065 | 11 (6.1)* | **4.288 (1.653-11.122), 0.003** |  |  |
| 4-10 days | 30 (34.9) | 26 (14.4)* | **7.273 (3.004-17.610), <0.001** | 14 (7.8)* | **8.920 (3.967-20.054), <0.001** |  |  |
| ≥11 days | 18 (20.9) | 16 (8.9)* | **4.818 (2.038-11.391), <0.001** | 7 (3.9)* | **11.525 (3.859-34.421), <0.001** |  |  |
| Trimethoprim/sulfamethoxazole (%) |  |  |  |  |  |  |  |
| 0 days | 63 (73.3) | 164 (91.1) | Reference | 169 (93.9) | Reference |  |  |
| 1-3 days | 4 (4.7) | 4 (2.2) | NA | 2 (1.1) | NA |  |  |
| 4-10 days | 8 (9.3) | 5 (2.8) | **5.726 (1.405-23.343), 0.015** | 4 (2.2) | NA |  |  |
| ≥11 days | 11 (12.8) | 7 (3.9) | **4.260 (1.500-12.095), 0.006** | 5 (2.8) | **5.158 (1.632-16.308), 0.005** |  |  |
| Vancomycin (%) |  |  |  |  |  |  |  |
| 0 days | 39 (45.3) | 144 (80.0) | Reference | 163 (90.6) | Reference |  |  |
| 1-3 days | 18 (20.9) | 13 (7.2) | **4.232 (1.900-9.426), <0.001** | 7 (3.9) | **6.991 (2.653-18.421), <0.001** |  |  |
| 4-10 days | 14 (16.3) | 20 (11.1) | **3.657 (1.379-9.698), 0.009** | 8 (4.4) | **6.079 (2.114-17.481), 0.001** |  |  |
| ≥11 days | 15 (17.4) | 3 (1.7) | NA | 2 (1.1) | NA |  |  |
| Selective digestive tract decontamination (%) |  |  |  |  |  |  |  |
| 0 days | 20 (23.3) | 86 (47.8)* | Reference | 138 (76.7) | Reference |  |  |
| 1-3 days | 10 (11.6) | 15 (8.3)* | 2.826 (0.918-8.704), 0.070 | 13 (7.2) | **7.507 (2.305-24.445), 0.001** |  |  |
| 4-10 days | 16 (18.6) | 40 (22.2)* | 1.748 (0.667-4.581), 0.256 | 18 (10.0) | **7.202 (2.541-20.412), <0.001** |  |  |
| ≥11 days | 40 (46.5) | 39 (21.7)* | **8.839 (3.254-24.009), <0.001** | 11 (6.1) | **26.012 (9.127-74.135), <0.001** |  |  |

Abbreviations: 95% CI= 95% confidence interval, OR= odds ratio, NA= not applicable, bold= statistically significant, *P*-value <0.05. *= included in multivariable analysis, **for 4 cases information about antibiotic use was missing.

**Table 2a.** Patient related clinical variables of case patients with a non-ICU department as ward of acquisition, univariate analysis

| Variables | Cases  (n= 54) | Controls 1&2 (n= 108) | Crude OR  (95% CI), *P*-value | Controls 3&4 (n= 108) | Crude OR  (95% CI), *P*-value |
| --- | --- | --- | --- | --- | --- |
| Basic characteristics |  |  |  |  |  |
| Age, years, median (range) | 57.5 (20-82) | 58.0 (19-92) | 1.004 (0.985-1.023), 0.710 | 57.2 (18-91) | 1.002 (0.981-1.023), 0.865 |
| Male gender (%) | 31 (57.4) | 56 (51.9) | 1.285 (0.640-2.579), 0.480 | 61 (56.5) | 1.039 (0.534-2.023), 0.910 |
| 28-day mortality (%) | 6 (11.1) | 15 (13.9) | 0.791 (0.301-2.084), 0.636 | 9 (8.3) | 1.363 (0.466-3.989), 0.572 |
| 1-year mortality (%) | 23 (42.6) | 30 (27.8) | 1.843 (0.944-3.596), 0.073 | 14 (13.0) | **4.363 (1.996-9.539), <0.001** |
| Transferred from another hospital (%) | 11 (20.4) | 18 (16.7) | 1.252 (0.565-2.777), 0.580 | 15 (13.9) | 1.585 (0.669-3.754), 0.295 |
| Length of admission, days, median (range) | 60 (8-223) | 23 (1-258) | **1.018 (1.008-1.027), <0.001** | 8 (1-302) | **1.048 (1.028-1.069), <0.001** |
| Erasmus MC 1y before VIM-PA (%) | 33 (61.1) | 51 (47.2)* | 1.803 (0.907-3.609), 0.093 | 42 (38.9)* | **2.948 (1.371-6.337), 0.006** |
| Erasmus MC ICU 1y before VIM-PA (%) | 8 (14.8) | 5 (4.6)* | **3.200 (1.047-9.782), 0.041** | 4 (3.7) | NA |
| Surgery (%) | 38 (70.4) | 26 (24.1)* | **7.055 (3.093-16.094), <0.001** | 39 (36.1)* | **5.432 (2.331-12.660), <0.001** |
| Underlying diseases |  |  |  |  |  |
| Cystic fibrosis (%) | 2 (3.7) | 4 (3.7) | NA | 1 (0.9) | NA |
| Chronic respiratory illness (%) | 11 (20.4) | 15 (13.9) | 1.707 (0.672-4.332), 0.261 | 13 (12.0) | 2.380 (0.823-6.882), 0.109 |
| Chronic kidney failure (%) | 2 (3.7) | 1 (0.9) | NA | 0 (0) | NA |
| Acute kidney failure; use of CVVH (%) | 3 (5.6) | 3 (2.8) | NA | 3 (2.8) | NA |
| Chronic liver failure (%) | 1 (1.9) | 5 (4.6) | NA | 7 (6.5) | NA |
| Acute liver failure (%) | 0 (0) | 2 (1.9) | NA | 1 (0.9) | NA |
| Chronic problems of the gastrointestinal tract (%) | 9 (16.7) | 9 (8.3) | 2.099 (0.802-5.490), 0.131 | 12 (11.1) | 1.673 (0.623-4.494), 0.308 |
| Acute problems of the gastrointestinal tract (%) | 9 (16.7) | 14 (13.0) | 1.319 (0.546-3.183), 0.538 | 21 (19.4) | 0.832 (0.356-1.946), 0.672 |
| Auto-immune disease (%) | 2 (3.7) | 7 (6.5) | NA | 8 (7.4) | NA |
| Human immunodeficiency virus (%) | 0 (0) | 0 (0) | NA | 2 (1.9) | NA |
| Diabetes (%) | 11 (20.4) | 17 (15.7) | 1.337 (0.596-3.002), 0.481 | 15 (13.9) | 1.674 (0.667-4.203), 0.272 |
| Solid organ transplant recipient (%) | 7 (13.0) | 8 (7.4) | 2.573 (0.623-10.621), 0.191 | 8 (7.4) | 1.832 (0.634-5.292), 0.264 |
| Stem cell/bone marrow transplant recipient (%) | 2 (3.7) | 3 (2.8) | NA | 1 (0.9) | NA |
| Use of immunosuppressive agents (%) | 18 (33.3) | 10 (9.3)* | **5.089 (1.994-12.989), 0.001** | 16 (14.8)* | **2.698 (1.253-5.809), 0.011** |
| Malignancies (%) | 15 (27.8) | 40 (37.0) | 0.585 (0.261-1.311), 0.193 | 32 (29.6) | 0.901 (0.414-1.961), 0.793 |
| Neutropenia, <500 cells/µL (%) | 1* (2.4) | 2** (3.0) | NA | 2*** (3.1) | NA |
| Endoscopies |  |  |  |  |  |
| Colonoscopy (%) | 2 (3.7) | 6 (5.6) | NA | 6 (5.6) | NA |
| Sigmoidoscopy (%) | 2 (3.7) | 1 (0.9) | NA | 3 (2.8) | NA |
| Endoscopic ultrasound (%) | 5 (9.3) | 13 (12.0) | 0.733 (0.240-2.239), 0.585 | 6 (5.6) | 2.257 (0.489-10.415), 0.297 |
| Gastroscopy (%) | 27 (50.0) | 25 (23.1)* | **5.043 (2.007-12.674), 0.001** | 21 (19.4)* | **5.925 (2.378-14.765), <0.001** |
| ERCP (%) | 6 (11.1) | 9 (8.3) | 1.399 (0.455-4.295), 0.558 | 4 (3.7) | NA |
| Bronchoscopy (%) | 10 (18.5) | 9 (8.3)* | 2.571 (0.958-6.905), 0.061 | 14 (13.0) | 1.511 (0.627-3.638), 0.357 |
| Transesophageal Echocardiography (TEE) (%) | 1 (1.9) | 6 (5.6) | NA | 5 (4.6) | NA |
| Medical devices |  |  |  |  |  |
| Mechanical ventilation (%) | 42 (77.8) | 55 (50.9)* | **5.049 (1.895-13.452), 0.001** | 55 (50.9)* | **5.882 (2.179-15.877), <0.001** |
| Tracheostomy (%) | 10 (18.5) | 9 (8.3)* | 2.701 (0.958-7.616), 0.060 | 4 (3.7) | NA |
| Extracorporeal membrane oxygenation (%) | 0 (0) | 0 (0) | NA | 0 (0) | NA |
| Central venous catheter (%) | 30 (55.6) | 28 (25.9)* | **3.627 (1.725-7.626), 0.001** | 20 (18.5)* | **6.215 (2.676-14.436), <0.001** |

Abbreviations: VIM-PA= Verona Integron-encoded Metallo-β-lactamase (VIM)-positive *Pseudomonas aeruginosa*, 95% CI= 95% confidence interval, OR= odds ratio, y= year, ICU= intensive care unit, CVVH= Continuous Veno-Venous Hemofiltration, ERCP= Endoscopic Retrograde Cholangiopancreatography, NA= not applicable. *For 12 patients information was missing. **for 42 patients information was missing, ***for 44 patients information was missing.

**Table 2b.** Treatment related variables of case patients with a non-ICU department as ward of acquisition, univariate analysis

| Variables | Cases**  (n= 52) | Controls 1&2 (n= 108) | Crude OR  (95% CI), *P*-value | Controls 3&4 (n= 108) | Crude OR  (95% CI), *P*-value |  |  |
| --- | --- | --- | --- | --- | --- | --- | --- |
| Treatment related variables; yes/no |  |  |  |  |  |  |  |
| Antifungals (%) | 24 (46.2) | 19 (17.6)* | **4.835 (2.023-11.552), <0.001** | 19 (17.6)* | **4.488 (1.969-10.229), <0.001** |  |  |
| Antivirals (%) | 4 (7.7) | 3 (2.8) | NA | 8 (7.4) | NA |  |  |
| Aminoglycosides (%) | 17 (32.7) | 10 (9.3) | **5.427 (1.967-14.968), 0.001** | 9 (8.3)* | **6.950 (2.299-21.016), 0.001** |  |  |
| Amoxicillin/clavulanic acid (%) | 15 (28.8) | 16 (14.8)* | 2.263 (0.990-5.172), 0.053 | 14 (13.0)* | **2.689 (1.147-6.307), 0.023** |  |  |
| Carbapenems (%) | 20 (38.5) | 19 (17.6)* | **3.358 (1.426-7.908), 0.006** | 8 (7.4)* | **8.790 (2.975-25.973), <0.001** |  |  |
| Cephalosporins (%) | 33 (63.5) | 22 (20.4)* | **6.019 (2.728-13.277), <0.001** | 31 (28.7) | **4.967 (2.211-11.158), <0.001** |  |  |
| Colistin (%) | 7 (13.5) | 6 (5.6)* | 3.562 (0.875-14.493), 0.076 | 5 (4.6)* | **5.622 (1.138-27.786), 0.034** |  |  |
| Macrolides (%) | 18 (34.6) | 8 (7.4)* | **4.948 (2.060-11.885), <0.001** | 6 (5.6)* | **8.293 (2.791-24.643), 0.001** |  |  |
| Metronidazole (%) | 16 (30.8) | 13 (12.0)* | **3.262 (1.365-7.798), 0.008** | 6 (5.6)* | **13.110), 2.979-57.701), 0.001** |  |  |
| Nitrofurantoin (%) | 6 (11.5) | 4 (3.7) | NA | 7 (6.5) | 1.804 (0.572-5.689), 0.314 |  |  |
| Penicillin (%) | 9 (17.3) | 18 (16.7) | 1.000 (0.412-2.426), 1.000 | 8 (7.4)* | 2.581 (0.901-7.392), 0.077 |  |  |
| Piperacillin/tazobactam (%) | 18 (34.6) | 20 (18.5) | **2.306 (1.053-5.052), 0.037** | 4 (3.7) | NA |  |  |
| Quinolones (%) | 34 (65.4) | 20 (18.5)* | **9.662 (3.717-25.119), <0.001** | 21 (19.4) | **15.042 (4.547-49.765), <0.001** |  |  |
| Trimethoprim/sulfamethoxazole (%) | 9 (17.3) | 7 (6.5)* | 2.788 (0.983-7.908), 0.054 | 8 (7.4)* | 2.788 (0.983-7.908), 0.054 |  |  |
| Vancomycin (%) | 27 (51.9) | 15 (13.9)* | **7.977 (3.023-21.047), <0.001** | 10 (9.3)* | **14.616 (4.394-48.620), <0.001** |  |  |
| Other antibiotics (%) | 12 (23.1) | 11 (10.2)* | **3.356 (1.133-9.937), 0.029** | 7 (6.5)* | **6.667 (1.835-24.224), 0.004** |  |  |
| Selective digestive tract decontamination (%) | 26 (50.0) | 15 (13.9) | **5.392 (2.408-12.072), <0.001** | 17 (15.7) | **7.353 (2.762-19.573), <0.001** |  |  |
| Treatment related variables; 3 categories | |  |  |  |  |  |  |
| Aminoglycosides (%) |  |  |  |  |  |  |  |
| 0 days | 35 (67.3) | 98 (90.7)* | Reference | 99 (91.7) | Reference |  |  |
| 1-3 days | 7 (13.5) | 5 (4.6)* | **4.192 (1.127-15.600), 0.033** | 3 (2.8) | NA |  |  |
| ≥4 days | 10 (19.2) | 5 (4.6)* | **6.952 (1.802-26.814), 0.005** | 6 (5.6) | **6.177 (1.640-23.260), 0.007** |  |  |
| Amoxicillin/clavulanic acid (%) |  |  |  |  |  |  |  |
| 0 days | 37 (71.2) | 92 (85.2) | Reference | 94 (87.0) | Reference |  |  |
| 1-3 days | 4 (7.7) | 5 (4.6) | NA | 7 (6.5) | NA |  |  |
| ≥4 days | 11 (21.2) | 11 (10.2) | 2.672 (0.951-7.512), 0.062 | 7 (6.5) | **3.964 (1.354-11.607), 0.012** |  |  |
| Carbapenems (%) |  |  |  |  |  |  |  |
| 0 days | 32 (61.5) | 89 (82.4) | Reference | 100 (92.6) | Reference |  |  |
| 1-3 days | 4 (7.7) | 6 (5.6) | NA | 4 (3.7) | NA |  |  |
| ≥4 days | 16 (30.8) | 13 (12.0) | **3.529 (1.444-8.625), 0.006** | 4 (3.7) | NA |  |  |
| Cephalosporins (%) |  |  |  |  |  |  |  |
| 0 days | 19 (36.5) | 86 (79.6) | Reference | 77 (71.3)* | Reference |  |  |
| 1-3 days | 12 (23.1) | 9 (8.3) | **5.445 (1.704-17.395), 0.004** | 14 (13.0)* | **3.859 (1.393-10.692), 0.009** |  |  |
| ≥4 days | 21 (40.4) | 13 (12.0) | **6.378 (2.485-16.370), 0.001** | 17 (15.7)* | **5.985 (2.316-15.467), <0.001** |  |  |
| Colistin (%) |  |  |  |  |  |  |  |
| 0 days | 45 (86.5) | 102 (94.4) | Reference | 103 (95.4) | Reference |  |  |
| 1-3 days | 2 (3.8) | 0 (0) | NA | 0 (0) | NA |  |  |
| ≥4 days | 5 (9.6) | 6 (5.6) | 2.667 (0.597-11.915), 0.199 | 5 (4.6) | 3.679 (0.682-19.862), 0.130 |  |  |
| Macrolides (%) |  |  |  |  |  |  |  |
| 0 days | 34 (65.4) | 100 (92.6) | Reference | 102 (94.4) | Reference |  |  |
| 1-3 days | 7 (13.5) | 1 (0.9) | NA | 2 (1.9) | NA |  |  |
| ≥4 days | 11 (21.2) | 7 (6.5) | **3.488 (1.266-9.390), 0.015** | 4 (3.7) | NA |  |  |
| Metronidazole (%) |  |  |  |  |  |  |  |
| 0 days | 36 (69.2) | 95 (88.0) | Reference | 102 (94.4) | Reference |  |  |
| 1-3 days | 6 (11.5) | 2 (1.9) | NA | 0 (0) | NA |  |  |
| ≥4 days | 10 (19.2) | 11 (10.2) | 2.408 (0.887-6.538), 0.085 | 6 (5.6) | **8.583 (1.855-39.714), 0.006** |  |  |
| Penicillin (%) |  |  |  |  |  |  |  |
| 0 days | 43 (82.7) | 90 (83.3) | Reference | 100 (92.6) | Reference |  |  |
| 1-3 days | 1 (1.9) | 2 (1.9) | NA | 4 (3.7) | NA |  |  |
| ≥4 days | 8 (15.4) | 16 (14.8) | 1.000 (0.385-2.596), 1.000 | 4 (3.7) | NA |  |  |
| Piperacillin/tazobactam (%) |  |  |  |  |  |  |  |
| 0 days | 34 (65.4) | 88 (81.5)* | Reference | 104 (96.3) | Reference |  |  |
| 1-3 days | 9 (17.3) | 7 (6.5)* | **3.606 (1.075-12.092), 0.038** | 2 (1.9) | NA |  |  |
| ≥4 days | 9 (17.3) | 13 (12.0)* | 1.654 (0.597-4.587), 0.333 | 2 (1.9) | NA |  |  |
| Quinolones (%) |  |  |  |  |  |  |  |
| 0 days | 18 (34.6) | 88 (81.5) | Reference | 87 (80.6) | Reference |  |  |
| 1-3 days | 6 (11.5) | 4 (3.7) | NA | 5 (4.6) | **13.827 (2.690-71.069), 0.002** |  |  |
| ≥4 days | 28 (53.8) | 16 (14.8) | **10.764 (3.802-30.475), <0.001** | 16 (14.8) | **15.275 (4.535-51.456), 0.001** |  |  |
| Trimethoprim/sulfamethoxazole (%) |  |  |  |  |  |  |  |
| 0 days | 43 (82.7) | 101 (93.5) | Reference | 100 (92.6) | Reference |  |  |
| 1-3 days | 3 (5.8) | 0 (0) | NA | 0 (0) | NA |  |  |
| ≥4 days | 6 (11.5) | 7 (6.5) | 2.000 (0.645-6.201), 0.230 | 8 (7.4) | **1.804 (0.572-5.689), 0.314** |  |  |
| Vancomycin (%) |  |  |  |  |  |  |  |
| 0 days | 25 (48.1) | 93 (86.1) | Reference | 98 (90.7) | Reference |  |  |
| 1-3 days | 3 (5.8) | 3 (2.8) | NA | 4 (3.7) | NA |  |  |
| ≥4 days | 24 (46.2) | 12 (11.1) | **12.093 (3.568-40.986), <0.001** | 6 (5.6) | **18.745 (5.081-69.159), <0.001** |  |  |
| Selective digestive tract decontamination (%) |  |  |  |  |  |  |  |
| 0 days | 26 (50.0) | 93 (86.1)* | Reference | 91 (84.3)* | Reference |  |  |
| 1-3 days | 7 (13.5) | 5 (4.6)* | **4.065 (1.194-13.841), 0.025** | 5 (4.6)* | **8.653 (1.793-41.760), 0.007** |  |  |
| ≥4 days | 19 (36.5) | 10 (9.3)* | **6.206 (2.401-16.038), <0.001** | 12 (11.1)* | **7.017 (2.492-19.758), <0.001** |  |  |
| Treatment related variables; 4 categories | | |  |  |  |  |  |
| Aminoglycosides (%) |  |  |  |  |  |  |  |
| 0 days | 35 (67.3) | 98 (90.7) | Reference | 99 (91.7) | Reference |  |  |
| 1-3 days | 7 (13.5) | 5 (4.6) | **4.208 (1.128-15.695), 0.032** | 3 (2.8) | NA |  |  |
| 4-10 days | 4 (7.7) | 2 (1.9) | NA | 3 (2.8) | NA |  |  |
| ≥11 days | 6 (11.5) | 3 (2.8) | NA | 3 (2.8) | NA |  |  |
| Amoxicillin/clavulanic acid (%) |  |  |  |  |  |  |  |
| 0 days | 37 (71.2) | 92 (85.2) | Reference | 94 (87.0) | Reference |  |  |
| 1-3 days | 4 (7.7) | 5 (4.6) | NA | 7 (6.5) | NA |  |  |
| 4-10 days | 9 (17.3) | 11 (10.2) | 2.235 (0.763-6.545), 0.142 | 6 (5.6) | **3.894 (1.178-12.866), 0.026** |  |  |
| ≥11 days | 2 (3.8) | 0 (0) | NA | 1 (0.9) | NA |  |  |
| Carbapenems (%) |  |  |  |  |  |  |  |
| 0 days | 32 (61.5) | 89 (82.4) | Reference | 100 (92.6) | Reference |  |  |
| 1-3 days | 4 (7.7) | 6 (5.6) | NA | 4 (3.7) | NA |  |  |
| 4-10 days | 7 (13.5) | 6 (5.6) | **3.381 (1.017-11.244), 0.047** | 2 (1.9) | NA |  |  |
| ≥11 days | 9 (17.3) | 7 (6.5) | **3.666 (1.164-11.550), 0.026** | 2 (1.9) | NA |  |  |
| Cephalosporins (%) |  |  |  |  |  |  |  |
| 0 days | 19 (36.5) | 86 (79.6) | Reference | 77 (71.3) | Reference |  |  |
| 1-3 days | 12 (23.1) | 9 (8.3) | **5.574 (1.718-18.088), 0.004** | 14 (13.0) | **3.995 (1.423-11.215), 0.009** |  |  |
| 4-10 days | 13 (25.0) | 7 (6.5) | **5.865 (2.086-16.488), 0.001** | 12 (11.1) | **4.912 (1.708-14.120), 0.003** |  |  |
| ≥11 days | 8 (15.4) | 6 (5.6) | **7.757 (1.916-31.402), 0.004** | 5 (4.6) | **9.308 (2.098-41.303), 0.003** |  |  |
| Colistin (%) |  |  |  |  |  |  |  |
| 0 days | 45 (86.5) | 102 (94.4) | Reference | 103 (95.4) | Reference |  |  |
| 1-3 days | 2 (3.8) | 0 (0) | NA | 0 (0) | NA |  |  |
| 4-10 days | 4 (7.7) | 2 (1.9) | NA | 3 (2.8) | NA |  |  |
| ≥11 days | 1 (1.9) | 4 (3.7) | NA | 2 (1.9) | NA |  |  |
| Macrolides (%) |  |  |  |  |  |  |  |
| 0 days | 34 (65.4) | 100 (92.6) | Reference | 102 (94.4) | Reference |  |  |
| 1-3 days | 7 (13.5) | 1 (0.9) | NA | 2 (1.9) | NA |  |  |
| 4-10 days | 7 (13.5) | 6 (5.6) | 2.611 (0.855-7.974), 0.092 | 4 (3.7) | NA |  |  |
| ≥11 days | 4 (7.7) | 1 (0.9) | NA | 0 (0) | NA |  |  |
| Metronidazole (%) |  |  |  |  |  |  |  |
| 0 days | 36 (69.2) | 95 (88.0) | Reference | 102 (94.4) | Reference |  |  |
| 1-3 days | 6 (11.5) | 2 (1.9) | **6.950 (1.357-35.590), 0.020** | 0 (0) | NA |  |  |
| 4-10 days | 6 (11.5) | 7 (6.5) | 2.130 (0.684-6.637), 0.192 | 4 (3.7) | NA |  |  |
| ≥11 days | 4 (7.7) | 4 (3.7) | NA | 2 (1.9) | NA |  |  |
| Penicillin (%) |  |  |  |  |  |  |  |
| 0 days | 43 (82.7) | 90 (83.3) | Reference | 100 (92.6) | Reference |  |  |
| 1-3 days | 1 (1.9) | 2 (1.9) | NA | 4 (3.7) | NA |  |  |
| 4-10 days | 4 (7.7) | 11 (10.2) | NA | 2 (1.9) | NA |  |  |
| ≥11 days | 4 (7.7) | 5 (4.6) | NA | 2 (1.9) | NA |  |  |
| Piperacillin/tazobactam (%) |  |  |  |  |  |  |  |
| 0 days | 34 (65.4) | 88 (81.5) | Reference | 104 (96.3) | Reference |  |  |
| 1-3 days | 9 (17.3) | 7 (6.5) | **3.627 (1.080-12.174), 0.037** | 2 (1.9) | NA |  |  |
| 4-10 days | 7 (13.5) | 9 (8.3) | 1.819 (0.601-5.508), 0.290 | 2 (1.9) | NA |  |  |
| ≥11 days | 2 (3.8) | 4 (3.7) | NA | 0 (0) | NA |  |  |
| Quinolones (%) |  |  |  |  |  |  |  |
| 0 days | 18 (34.6) | 88 (81.5) | Reference | 87 (80.6)* | Reference |  |  |
| 1-3 days | 6 (11.5) | 4 (3.7) | NA | 5 (4.6)* | **13.528 (2.597-70.469), 0.002** |  |  |
| 4-10 days | 13 (25.0) | 11 (10.2) | **6.501 (2.153-19.627), 0.001** | 6 (5.6)* | **16.352 (3.857-69.338), <0.001** |  |  |
| ≥11 days | 15 (28.8) | 5 (4.6) | **36.094 (6.755-192.871), <0.001** | 10 (9.3)* | **14.394 (3.619-57.258), <0.001** |  |  |
| Trimethoprim/sulfamethoxazole (%) |  |  |  |  |  |  |  |
| 0 days | 43 (82.7) | 101 (93.5) | Reference | 100 (92.6) | Reference |  |  |
| 1-3 days | 3 (5.8) | 0 (0) | NA | 0 (0) | NA |  |  |
| 4-10 days | 3 (5.8) | 3 (2.8) | NA | 0 (0) | NA |  |  |
| ≥11 days | 3 (5.8) | 4 (3.7) | NA | 8 (7.4) | NA |  |  |
| Vancomycin (%) |  |  |  |  |  |  |  |
| 0 days | 25 (48.1) | 93 (86.1) | Reference | 98 (90.7) | Reference |  |  |
| 1-3 days | 3 (5.8) | 3 (2.8) | NA | 4 (3.7) | NA |  |  |
| 4-10 days | 9 (17.3) | 6 (5.6) | **7.728 (1.927-30.981), 0.004** | 3 (2.8) | NA |  |  |
| ≥11 days | 15 (28.8) | 6 (5.6) | **21.658 (4.076-115.077), 0.001** | 3 (2.8) | NA |  |  |
| Selective digestive tract decontamination (%) |  |  |  |  |  |  |  |
| 0 days | 26 (50.0) | 93 (86.1) | Reference | 91 (84.3) | Reference |  |  |
| 1-3 days | 7 (13.5) | 5 (4.6) | **3.967 (1.158-13.591), 0.028** | 5 (4.6) | **7.625 (1.597-36.403), 0.011** |  |  |
| 4-10 days | 4 (7.7) | 7 (6.5) | NA | 7 (6.5) | NA |  |  |
| ≥11 days | 15 (28.8) | 3 (2.8) | NA | 5 (4.6) | **10.731 (3.145-36.608), <0.001** |  |  |

Abbreviations: 95% CI= 95% confidence interval, OR= odds ratio, NA= not applicable. Bold= statistically significant, *P*-value <0.05. *= included in multivariable analysis, ** for two cases information about antibiotic use was missing.
