# Supplementary material for: VIM-positive Pseudomonas aeruginosa in a large tertiary care hospital: matched case-control studies and a network analysis
Source: Antimicrob Resist Infect Control. 2018 Feb 27;7:32. doi: 10.1186/s13756-018-0325-1 (PMC5828133; doi:10.1186/s13756-018-0325-1)
Supplement: Supplementary file 6 — Text file: Crude odds ratios; control group 1&2 versus control group 3&4. Variables with an asterisk (*) were selected for multivariable analysis. (DOCX 35 kb) [file 13756_2018_325_MOESM6_ESM.docx]

**Additional file 6: Text file:** Crude odds ratios; control group 1&2 versus control group 3&4.

| Variables | Controls 1&2 (n= 288) | Controls 3&4 (n= 288) | Crude OR  (95% CI), P-value |
| --- | --- | --- | --- |
| Basic characteristics |  |  |  |
| Age, years, median (range) | 60.3 (17-92) | 61.1 (17-91) | 0.997 (0.987-1.007), 0.591 |
| Male gender (%) | 174 (60.4) | 167 (58.0) | 1.095 (0.799-1.500), 0.574 |
| 28-day mortality (%)*,** | **69 (24.0)** | **40 (13.9)** | **2.208 (1.363-3.577), 0.001** |
| 1-year mortality (%)*,** | **95 (33.0)** | **63 (21.9)** | **1.780 (1.215-2.610), 0.003** |
| Transferred from another hospital (%) | 60 (20.8) | 50 (17.4) | 1.227 (0.824-1.827), 0.313 |
| Median length of admission (range)* | **25 (1-258)** | **12 (1-1102)** | **1.007 (1.002-1.013), 0.010** |
| Erasmus MC 1y before VIM-PA (%)** | 111 (38.5) | 109 (37.8) | 1.030 (0.736-1.441), 0.864 |
| Erasmus MC ICU 1y before VIM-PA (%)** | 15 (5.2) | 14 (4.9) | 1.071 (0.517-2.220), 0.853 |
| Surgery (%) | 119 (41.3) | 121 (42.0) | 0.971 (0.694-1.359), 0.864 |
| Underlying diseases |  |  |  |
| Cystic fibrosis (%) | 3 (1.0) | 2 (0.7) | 1.500 (0.251-8.977), 0.657 |
| Chronic respiratory illness (%) | 43 (14.9) | 43 (14.9) | 1.000 (0.608-1.645), 1.000 |
| Chronic kidney failure (%)* | **3 (1.0)** | **11 (3.8)** | **0.273 (0.076-0.978), 0.046** |
| Acute kidney failure; use of CVVH (%) | 18 (6.3) | 10 (3.5) | 1.889 (0.842-4.237), 0.123 |
| Chronic liver failure (%)* | **7 (2.4)** | **17 (5.9)** | **0.412 (0.171-0.993), 0.048** |
| Acute liver failure (%) | 6 (2.1) | 3 (1.0) | 2.000 (0.500-7.997), 0.327 |
| Chronic problems of the gastrointestinal tract (%) | 28 (9.7) | 26 (9.0) | 1.100 (0.600-2.015), 0.758 |
| Acute problems of the gastrointestinal tract (%)* | 39 (13.5) | 54 (18.8) | 0.605 (0.361-1.016), 0.057 |
| Auto-immune disease (%) | 18 (6.3) | 26 (9.0) | 0.667 (0.354-1.255), 0.209 |
| Human immunodeficiency virus (%) | 2 (0.7) | 4 (1.4) | 0.500 (0.092-2.730), 0.423 |
| Diabetes (%) | 37 (12.8) | 48 (16.7) | 0.725 (0.450-1.169), 0.187 |
| Solid organ transplant recipient (%) | 20 (6.9) | 30 (10.4) | 0.643 (0.356-1.162), 0.144 |
| Stem cell/bone marrow transplant recipient (%)* | 6 (2.1) | 1 (0.3) | 6.000 (0.722-49.837), 0.097 |
| Use of immunosuppressive agents (%)* | 71 (24.7) | 55 (19.1) | 1.444 (0.944-2.209), 0.090 |
| Malignancies (%) | 76 (26.4) | 92 (31.9) | 0.729 (0.492-1.080), 0.115 |
| Neutropenia, <500 cells/µL (%) | 4* (2.1) | 7* (2.4) | 0.750 (0.168-3.351), 0.706 |
| Endoscopies |  |  |  |
| Colonoscopy (%) | 10 (3.5) | 12 (4.2) | 0.833 (0.360-1.929), 0.670 |
| Sigmoidoscopy (%) | 5 (1.7) | 8 (2.8) | 0.571 (0.167-1.952), 0.372 |
| Endoscopic ultrasound (%) | 26 (9.0) | 19 (6.6) | 1.412 (0.758-2.628), 0.277 |
| Gastroscopy (%) | 50 (17.4) | 59 (20.5) | 0.804 (0.522-1.240), 0.324 |
| ERCP (%) | 15 (5.2) | 10 (3.5) | 1.625 (0.674-3.921), 0.280 |
| Bronchoscopy (%) | 34 (11.8) | 33 (11.5) | 1.037 (0.611-1.759), 0.893 |
| Transesophageal Echocardiography (TEE) (%) | 13 (4.5) | 13 (4.5) | 1.000 (0.464-2.157), 1.000 |
| Medical devices |  |  |  |
| Mechanical ventilation (%)* | **210 (72.9)** | **185 (64.2)** | **1.735 (1.138-2.646), 0.010** |
| Tracheostomy (%)* | **38 (13.2)** | **9 (3.1)** | **5.143 (2.289-11.556), <0.001** |
| Extracorporeal membrane oxygenation (%) | 0 (0) | 0 (0) | NA |
| Central venous catheter (%)* | **124 (43.1)** | **77 (26.7)** | **2.382 (1.596-3.556), <0.001** |
| Treatment related variables; yes/no |  |  |  |
| Antifungals (%)* | **79 (28.0)** | **50 (17.7)** | **1.967 (1.267-3.052), 0.003** |
| Antivirals (%) | 19 (6.7) | 17 (6.0) | 1.125 (0.574-2.206), 0.732 |
| Aminoglycosides (%) | **53 (18.8)** | **24 (8.5)** | **2.450 (1.456-4.121), 0.001** |
| Amoxicillin/clavulanic acid (%) | 48 (17.0) | 40 (14.2) | 1.242 (0.786-1.965), 0.353 |
| Carbapenems (%) | **58 (20.7)** | **26 (9.2)** | **2.600 (1.552-4.355), <0.001** |
| Cephalosporins (%)* | **131 (46.5)** | **107 (37.9)** | **1.632 (1.090-2.443), 0.017** |
| Colistin (%) | 20 (7.1) | 12 (4.3) | 1.800 (0.831-3.899), 0.136 |
| Macrolides (%)* | **64 (22.7)** | **27 (9.6)** | **2.762 (1.677-4.550), <0.001** |
| Metronidazole (%)* | **48 (17.0)** | **32 (11.3)** | **1.762 (1.031-3.010), 0.038** |
| Nitrofurantoin (%)* | **12 (4.3)** | **24 (8.5)** | **0.400 (0.176-0.908), 0.028** |
| Penicillin (%) | **49 (17.4)** | **31 (11.0)** | **1.692 (1.042-2.748), 0.033** |
| Piperacillin/tazobactam (%)* | **50 (17.7)** | **32 (11.3)** | **1.818 (1.081-3.059), 0.024** |
| Quinolones (%)* | **88 (31.2)** | **53 (18.8)** | **2.129 (1.389-3.262), 0.001** |
| Trimethoprim/sulfamethoxazole (%) | 23 (8.2) | 19 (6.7) | 1.286 (0.639-2.585), 0.481 |
| Vancomycin (%)* | **51 (18.1)** | **27 (9.6)** | **2.143 (1.277-3.597), 0.004** |
| Other antibiotics (%) | 28 (9.9) | 30 (10.6) | 0.923 (0.530-1.608), 0.777 |
| Selective digestive tract decontamination (%) | **109 (38.7)** | **59 (20.9)** | **2.786 (1.809-4.290), <0.001** |
| Treatment related variables; 3 categories |  |  |  |
| Aminoglycosides (%) |  |  |  |
| 0 days | 235 (81.6) | 264 (91.7) | Reference |
| 1-3 days | 24 (8.3) | 7 (2.4) | **3.514 (1.511-8.169), 0.004** |
| ≥4 days | 29 (10.1) | 17 (5.9) | 1.921 (0.999-3.691), 0.050 |
| Amoxicillin/clavulanic acid (%) |  |  |  |
| 0 days | 240 (83.3) | 248 (86.1) | Reference |
| 1-3 days | 18 (6.2) | 18 (6.2) | 1.040 (0.539-2.007), 0.907 |
| ≥4 days | 30 (10.4) | 22 (7.6) | 1.426 (0.791-2.572), 0.238 |
| Carbapenems (%)* |  |  |  |
| 0 days | 230 (79.9) | 262 (91.0) | Reference |
| 1-3 days | 16 (5.6) | 9 (3.1) | 1.875 (0.795-4.422), 0.151 |
| ≥4 days | 42 (14.6) | 17 (5.9) | **3.083 (1.608-5.913), 0.001** |
| Cephalosporins (%) |  |  |  |
| 0 days | 157 (54.5) | 181 (62.8) | Reference |
| 1-3 days | 41 (14.2) | 51 (17.7) | 1.050 (0.604-1.827), 0.862 |
| ≥4 days | 90 (31.3) | 56 (19.4) | **1.976 (1.270-3.076), 0.003** |
| Colistin (%) |  |  |  |
| 0 days | 268 (93.1) | 276 (95.8) | Reference |
| 1-3 days | 4 (1.4) | 1 (0.3) | 4.435 (0.488-40.316), 0.186 |
| ≥4 days | 16 (5.6) | 11 (3.8) | 1.591 (0.707-3.581), 0.262 |
| Macrolides (%) |  |  |  |
| 0 days | 224 (77.8) | 261 (90.6) | Reference |
| 1-3 days | 30 (10.4) | 11 (3.8) | **3.150 (1.522-6.516), 0.002** |
| ≥4 days | 34 (11.8) | 16 (5.6) | **2.489 (1.316-4.708), 0.005** |
| Metronidazole (%) |  |  |  |
| 0 days | 240 (83.3) | 256 (88.9) | Reference |
| 1-3 days | 14 (4.9) | 12 (4.2) | 1.400 (0.613-3.199), 0.424 |
| ≥4 days | 34 (11.8) | 20 (6.9) | **1.963 (1.057-3.643), 0.033** |
| Penicillin (%)* |  |  |  |
| 0 days | 239 (83.0) | 257 (89.2) | Reference |
| 1-3 days | 12 (4.2) | 14 (4.9) | 0.903 (0.402-2.028), 0.805 |
| ≥4 days | 37 (12.8) | 17 (5.9) | **2.324 (1.268-4.259), 0.006** |
| Piperacillin/tazobactam (%) |  |  |  |
| 0 days | 238 (82.6) | 256 (88.9) | Reference |
| 1-3 days | 22 (7.6) | 15 (5.2) | 1.787 (0.835-3.822), 0.135 |
| ≥4 days | 28 (9.7) | 17 (31.3) | 1.840 (0.967-3.499), 0.063 |
| Quinolones (%) |  |  |  |
| 0 days | 200 (69.4) | 235 (81.6) | Reference |
| 1-3 days | 30 (10.4) | 16 (5.6) | **2.373 (1.208-4.660), 0.012** |
| ≥4 days | 58 (20.1) | 37 (12.8) | **2.017 (1.227-3.314), 0.006** |
| Trimethoprim/sulfamethoxazole (%) |  |  |  |
| 0 days | 265 (92.0) | 269 (93.4) | Reference |
| 1-3 days | 4 (1.4) | 2 (0.7) | 2.058 (0.375-11.302), 0.406 |
| ≥4 days | 19 (6.6) | 17 (5.9) | 1.184 (0.561-2.497), 0.658 |
| Vancomycin (%) |  |  |  |
| 0 days | 237 (82.3) | 261 (90.6) | Reference |
| 1-3 days | 16 (5.6) | 11 (3.8) | 1.685 (0.723-3.926), 0.227 |
| ≥4 days | 35 (12.2) | 16 (5.6) | **2.415 (1.289-4.522), 0.006** |
| Selective digestive tract decontamination (%)* |  |  |  |
| 0 days | 179 (62.2) | 229 (79.5) | Reference |
| 1-3 days | 20 (6.9) | 18 (6.3) | 1.858 (0.895-3.854), 0.096 |
| ≥4 days | 89 (30.9) | 41 (14.2) | **3.075 (1.939-4.877), <0.001** |
| Treatment related variables; 4 categories |  |  |  |
| Aminoglycosides (%)* |  |  |  |
| 0 days | 235 (81.6) | 264 (91.7) | Reference |
| 1-3 days | 24 (8.3) | 7 (2.4) | **3.543 (1.522-8.249), 0.003** |
| 4-10 days | 18 (6.2) | 9 (3.1) | 2.328 (0.992-5.462), 0.052 |
| ≥11 days | 11 (3.8) | 8 (2.8) | 1.505 (0.598-3.788), 0.385 |
| Amoxicillin/clavulanic acid (%) |  |  |  |
| 0 days | 240 (83.3) | 248 (86.1) | Reference |
| 1-3 days | 18 (6.2) | 18 (6.2) | 1.050 (0.544-2.029), 0.883 |
| 4-10 days | 29 (10.1) | 19 (6.6) | 1.565 (0.849-2.887), 0.151 |
| ≥11 days | 1 (0.3) | 3 (1.0) | 0.369 (0.038-3.588), 0.391 |
| Carbapenems (%) |  |  |  |
| 0 days | 230 (79.9) | 262 (91.0) | Reference |
| 1-3 days | 16 (5.6) | 9 (3.1) | 1.875 (0.795-4.422), 0.151 |
| 4-10 days | 26 (9.0) | 12 (4.2) | **2.535 (1.202-5.346), 0.015** |
| ≥11 days | 16 (5.6) | 5 (1.7) | **5.006 (1.426-17.573), 0.012** |
| Cephalosporins (%) |  |  |  |
| 0 days | 157 (54.5) | 181 (62.8) | Reference |
| 1-3 days | 41 (14.2) | 51 (17.7) | 1.034 (0.594-1.802), 0.905 |
| 4-10 days | 70 (24.3) | 46 (16.0) | **1.858 (1.156-2.987), 0.011** |
| ≥11 days | 20 (6.9) | 10 (3.5) | **2.505 (1.103-5.688), 0.028** |
| Colistin (%) |  |  |  |
| 0 days | 268 (93.1) | 276 (95.8) | Reference |
| 1-3 days | 4 (1.4) | 1 (0.3) | 4.086 (0.451-37.066), 0.211 |
| 4-10 days | 9 (3.1) | 4 (1.4) | 2.250 (0.693-7.306), 0.177 |
| ≥11 days | 7 (2.4) | 7 (2.4) | 1.110 (0.349-3.526), 0.860 |
| Macrolides (%) |  |  |  |
| 0 days | 224 (77.8) | 261 (90.6) | Reference |
| 1-3 days | 30 (10.4) | 11 (3.8) | **3.111 (1.506-6.429), 0.002** |
| 4-10 days | 28 (9.7) | 15 (5.2) | **2.202 (1.119-4.332), 0.022** |
| ≥11 days | 6 (2.1) | 1 (0.3) | 6.000 (0.722-49.837), 0.097 |
| Metronidazole (%) |  |  |  |
| 0 days | 240 (83.3) | 256 (88.9) | Reference |
| 1-3 days | 14 (4.9) | 12 (4.2) | 1.394 (0.608-3.193), 0.432 |
| 4-10 days | 25 (8.7) | 18 (6.2) | 1.628 (0.840-3.154), 0.149 |
| ≥11 days | 9 (3.1) | 2 (0.7) | **5.132 (1.091-24.136), 0.038** |
| Penicillin (%) |  |  |  |
| 0 days | 239 (83.0) | 257 (89.2) | Reference |
| 1-3 days | 12 (4.2) | 14 (4.9) | 0.888 (0.396-1.990), 0.773 |
| 4-10 days | 25 (8.7) | 14 (4.9) | 1.835 (0.933-3.608), 0.079 |
| ≥11 days | 12 (4.2) | 3 (1.0) | **5.500 (1.219-24.813), 0.027** |
| Piperacillin/tazobactam (%) |  |  |  |
| 0 days | 238 (82.6) | 256 (88.9) | Reference |
| 1-3 days | 22 (7.6) | 15 (5.2) | 1.807 (0.842-3.877), 0.129 |
| 4-10 days | 20 (6.9) | 13 (4.5) | 1.695 (0.817-3.517), 0.156 |
| ≥11 days | 8 (2.8) | 4 (1.4) | 2.328 (0.687-7.885), 0.175 |
| Quinolones (%) |  |  |  |
| 0 days | 200 (69.4) | 235 (81.6) | Reference |
| 1-3 days | 30 (10.4) | 16 (5.6) | **2.452 (1.241-4.847), 0.010** |
| 4-10 days | 37 (12.8) | 20 (6.9) | **2.556 (1.352-4.833), 0.004** |
| ≥11 days | 21 (7.3) | 17 (5.9) | 1.500 (0.762-2.953), 0.240 |
| Trimethoprim/sulfamethoxazole (%) |  |  |  |
| 0 days | 265 (92.0) | 269 (93.4) | Reference |
| 1-3 days | 4 (1.4) | 2 (0.7) | 2.371 (0.414-13.563), 0.332 |
| 4-10 days | 8 (2.8) | 4 (1.4) | 2.533 (0.635-10.105), 0.188 |
| ≥11 days | 11 (3.8) | 13 (4.5) | 0.853 (0.352-2.070), 0.726 |
| Vancomycin (%) |  |  |  |
| 0 days | 237 (82.3) | 261 (90.6) | Reference |
| 1-3 days | 16 (5.6) | 11 (3.8) | 1.705 (0.730-3.985), 0.218 |
| 4-10 days | 26 (9.0) | 11 (3.8) | **2.764 (1.280-5969), 0.010** |
| ≥11 days | 9 (3.1) | 5 (1.7) | 1.800 (0.603-5.371), 0.292 |
| Selective digestive tract decontamination (%) |  |  |  |
| 0 days | 179 (62.2) | 229 (79.5) | Reference |
| 1-3 days | 20 (6.9) | 18 (6.2) | 1.891 (0.908-3.938), 0.089 |
| 4-10 days | 47 (16.3) | 25 (8.7) | **2.636 (1.524-4.560), 0.001** |
| ≥11 days | 42 (14.6) | 16 (5.6) | **3.820 (1.997-7.305), <0.001** |

Abbreviations: VIM-PA= Verona Integron-encoded Metallo-β-lactamase (VIM)-positive *Pseudomonas aeruginosa*, 95% CI= 95% confidence interval, OR= odds ratio, y= year, ICU= intensive care unit, CVVH= Continuous Veno-Venous Hemofiltration, ERCP= Endoscopic Retrograde Cholangiopancreatography, NA= not applicable, *= used in multivariable analysis, **= based on the date of identification of VIM-PA of the case patient to who the control was matched.
